# *Staphylococcus aureus* characterization in commercial rabbit farms reveals high genetic diversity and widespread antimicrobial resistance

**DOI:** 10.3389/fvets.2025.1673809

**Published:** 2025-10-30

**Authors:** Patricia Mascarós, Carmen Martínez-Seijas, José Francisco Díaz-Méndez, Juan María Rosell Pujol, Celia Sanz, Alberto Arnau-Bonachera, Laura Selva, Juan M. Corpa, David Viana

**Affiliations:** ^1^Pathology Group, Departamento Producción y Sanidad Animal, Salud Pública Veterinaria y Ciencia y Tecnología de los Alimentos, Facultad de Veterinaria, Universidad Cardenal Herrera-CEU, CEU Universities, Valencia, Spain; ^2^Cunivet Service, Tarragona, Spain; ^3^Veterinary Diagnostic Disease and Autogenous Vaccine Laboratory, Exopol SL, Zaragoza, Spain

**Keywords:** livestock-associated *Staphylococcus aureus*, epidemiological surveillance, clonal diversity, geographic dissemination, antimicrobial resistance, methicillin-resistant *staphylococcus aureus*, multidrug resistance

## Abstract

*Staphylococcus aureus* is a relevant bacterium in animals and a major public health concern, recognized by the World Health Organization as a priority pathogen due to its critical role in antimicrobial resistance. In rabbits, it is a leading cause of mastitis, pododermatitis, and abscesses, and the primary cause of culling in reproductive does. However, its genetic diversity, transmission dynamics, and resistance patterns in this context remain poorly understood. This study aimed to characterize the genetic structure, geographical dissemination, and antimicrobial resistance profiles of *S. aureus* isolates from rabbit farms across the Iberian Peninsula. A sampling strategy based on the official farm census ensured representativeness. Sequencing and epidemiological data were used to assess clonal lineages and distribution, and antimicrobial resistance was also evaluated. The results revealed high clonal diversity, including 10 clonal complexes (CCs) and 35 sequence types, with 18 previously unreported in rabbits. However, this diversity was largely dominated by two lineages, CC121 and CC96, which together account for nearly 90% of all isolates. Geographical proximity and commerce influenced strain distribution, with defined patterns for certain lineages. Resistance was observed against all 14 tested antibiotics, including key classes for human medicine: cephalosporins, oxazolidinones, and glycylcyclines. Notably, 86.1% of isolates were classified as multidrug-resistant (MDR), and 9.1% were methicillin-resistant (MRSA). This study provides the most comprehensive and representative characterization to date of *S. aureus* diversity, dissemination patterns, and antimicrobial resistance in rabbit farming, underscoring the importance of continuous surveillance and reinforcing the need for improved control strategies and preventive measures within a One Health framework. To support the rabbit industry, we have developed “StaphyMAP,” an open-access, interactive platform that provides anonymized, real-time data on the distribution, genetic diversity, and antimicrobial resistance patterns of *Staphylococcus aureus* isolates. This tool aims to assist veterinary clinicians in making informed empirical treatment decisions, thereby promoting more rational antimicrobial use and helping to curb the overuse of broad-spectrum antibiotics.

## 1 Introduction

*Staphylococcus aureus* is an opportunistic pathogen of major relevance in both human and veterinary medicine, responsible for a wide range of infections in multiple animal species (1). In livestock production, its presence represents a significant health and economic challenge, particularly in intensive systems, where it can cause recurrent outbreaks and reduce productive performance. In rabbit farming, *S. aureus* is a major etiological agent of cutaneous and mammary infections (2) with clinical signs reported in more than 60% of farms (3). These infections directly affect animal welfare and farm profitability, as one of the leading causes of culling and mortality (4, 5). The scarcity of data on *S. aureus* in rabbit production systems, particularly regarding its clonality, resistance trends, and circulation dynamics, raises growing concern, particularly due to increasingly frequent reports of highly virulent and drug-resistant strains from clinic veterinarians.

Genetic diversity is a key factor in understanding the epidemiology of *S. aureus* infections and in designing effective control and prevention strategies, which can be better understood through the analysis of strain genomes and their genetic relatedness (6). In this regard, several studies have aimed to characterize *S. aureus* clones in rabbitries. Strains of clonal complex (CC) 121 have consistently been reported as the most frequent and virulent in rabbits (7–10), followed by CC96, which is considered less virulent but remains the second most frequently isolated lineage (7, 8, 11). The most extensive study conducted in the Iberian Peninsula (Spain and Portugal) to date identified several sequence types not previously reported in rabbits (7), suggesting that earlier sampling efforts may have underestimated clonal diversity. However, previous studies conducted with isolates obtained from rabbits had mainly relied on virulence and phage profiling (12–17), without a genomic characterization with multi-locus sequence typing (MLST) until after 2006 (9), limiting the comparability of the results with current methods. In addition, most available studies which did apply MLST typing are either geographically restricted and based on smaller sample sizes, or focused on virulence models using pre-sampled isolates or characterizing them conveniently to present their results (5, 8, 9, 18–21), restricting a broader understanding of the pathogen’s epidemiology. Therefore, key questions remain unanswered regarding the origin, genetic stability, and dissemination routes of certain strains, as no integrative studies combining genomic and epidemiological approaches have been conducted to date.

Moreover, antibiotic resistance in rabbitries has emerged as a growing concern. Resistance profiles in rabbit-derived strains have been increasingly studied in recent years. While early studies reported predominantly susceptible isolates (15), more recent works have shown a progressive rise in resistance rates (22–24). The most recent work conducted in the Iberian Peninsula reported a significant increase in resistance to macrolides, tetracyclines, and aminoglycosides (25), most of the main antimicrobial classes used to treat staphylococcal infections in rabbits. However, because the analysis was restricted to methicillin-resistant isolates, generalizing these findings to the population level may overestimate resistance frequencies to other antimicrobial classes (26–29).

In this context, there is a need for a comprehensive study capable of providing a representative description of *S. aureus* populations in commercial rabbit farms, assessing not only their genetic diversity and geographical distribution, but also global antimicrobial resistance patterns and their transmission potential. This study addresses that need through a sampling design based on an official census of rabbit farms, ensuring the generation of representative data combining genomic and epidemiological approaches to examine strain diversity.

It is hypothesized that a few well-adapted clones play a dominant role in the epidemiology of the pathogen, although the overall genetic diversity of *S. aureus* in this host may be underestimated and hidden transmission routes may contribute to its persistence and spread. The aim of this study is to characterize the genetic structure, distribution patterns, and antimicrobial resistance profiles of *S. aureus* in commercial rabbit farming across the Iberian Peninsula. Specifically, the study seeks to: (I) describe the clonal structure and geographic distribution of circulating strains; (II) analyze genomic distances between isolates to assess genetic relationships and explore their potential association with geographic distribution; (III) develop an open-access platform (StaphyMAP) to facilitate real-time surveillance for the rabbit industry; and (IV) explore antibiotic resistance patterns to support the development of effective control strategies.

## 2 Materials and methods

### 2.1 Population description and sampling

To obtain a complete overview of the health status of commercial rabbit farms regarding staphylococcal infections, a nationwide sampling strategy was designed, based on the geographical distribution of the rabbit farming census in Spain. This approach, based on data provided by the Spanish Ministry of Agriculture, Fisheries and Food (2020), ensured a representative and unbiased sampling. The sample size was calculated using a stochastic simulation, with the aim of ensuring that significant differences could be detected between two communities whenever the observed difference was greater than 5–10%. A total of 50 farms per autonomous community were to be sampled, with at least two samples collected from each farm. In communities with fewer than 50 farms, all available farms were to be included. Farms within each autonomous community were sampled randomly. To implement this strategy, collaborations were established with the Association of Veterinarians Specialized in Rabbit Production (AVECU), the interprofessional organization INTERCUN, and the private clinical laboratory Exopol (San Mateo de Gállego, Zaragoza), which facilitated access to samples and farms distributed throughout the country. On each farm, a minimum of two animals presenting lesions compatible with staphylococcal infections (purulent mastitis, abscesses, pododermatitis and other suppurative lesions) were sampled. Sterile swabs were taken directly from the suspected lesions and sent to the laboratory under refrigeration at ≤4 °C, along with a detailed report including information on the animal, the farm, and the clinical characteristics of the outbreak. The sampled animals included both males and females, ranging in age from 7 days old to adults. Sampling was conducted between November 2021 and February 2023. Using this sampling strategy, swabs were collected from 39 out of 50 Spanish provinces, across 14 out of 17 autonomous communities, as well as from five districts in northern Portugal, which were grouped and treated as a single territorial unit for analysis. A total of 285 farms were analyzed, obtaining 783 samples.

### 2.2 *Staphylococcus aureus* isolation

Samples were inoculated on Columbia CNA (colistin-nalidixic acid) Agar with 5% Sheep Blood (Becton-Dickinson, Sparks, MD, USA) and incubated at 37 °C for 24 h aerobically. Colonies were selected by morphological and hemolytic characteristics (30), including round colonies, from transparent-white to golden in color, with and without hemolysis. As a follow-up test, colonies meeting the criteria, were then inoculated on CHROMagar™ Staph aureus and CHROMagar™ Orientation. Compatible colonies were then grown in Tryptic Soy Broth (TSB; Difco, Becton Dickinson, Sparks, MD, USA) for 16 h with shaking at 37 °C and were then stored at −80 °C.

### 2.3 Molecular typing and sequencing

The Genelute Bacterial Genomic DNA kit (Sigma-Aldrich, St. Louis, MO, USA) was used to extract DNA. To do so, the manufacturer’s instructions were followed, incorporating 1 μL of lysostaphin (10 mg/mL, Sigma-Aldrich) to lyse bacterial cells at 37 °C for 1 h prior to the extraction.

The identification of *S. aureus* was confirmed by PCR amplification of the *coa* (coagulase) and *spa* (protein A) genes. These same genes, together with *clf*B (clumping factor B), were then used to genotype the isolates, by amplifying their polymorphic regions, as previously described (18). Expected values for clonal complexes (CCs) and sequence types (STs) were then inferred, based on previous work (7). A stratified sampling approach with proportional allocation, based on the genotypes observed in our study population, was used to select strains for whole genome sequencing.

Two hundred and forty-three samples were sequenced on a Hiseq XTen (Illumina) (2 × 150 bp paired-end). DNA libraries were prepared with xGen DNA Library Prep EZ (IDT). Capillary electrophoresis was carried out using the QIAxcel Advanced System (Qiagen) to assess the output of libraries. Preprocessing was applied to raw sequences using *FastP* v0.23.2 (31) and *FastQC* (32). Assembly was using *Shovill* 0.7.1 (33), followed by quality assessment using *Quast* (34). *Fast MLST* (35) was used to obtain MLST profiles.

The absolute error between the expected CCs and STs (inferred from genotypes) and the observed ones (obtained through sequencing) was calculated to evaluate the strength of the correlation and determine whether genotypes could be reliably used to infer the MLST profiles without the need for sequencing. Consequently, for each unsequenced sample, the most likely CC and ST was assigned based on their corresponding genotypes. Once a sample from a specific farm had been sequenced, the ST obtained for that isolate was assigned to all unsequenced isolates from the same farm, with the same genotype. In cases where multiple STs were equally probable for a given genotype, the sample was classified as “undefined,” and no inference was performed. As a result, CCs and STs were assigned to all isolates included in this study.

### 2.4 Mapping

The maps illustrating the geographical distribution of *S. aureus* isolates were generated using the free software R Version 2023.12.0 + 369 and the following R packages: *sf*, *ggplot2*, *readxl*, *dplyr* and *svglite*. Geospatial data was obtained from the *GitHub* repository (36).

The distribution of CCs was analyzed separately for each one of the sampled autonomous communities of Spain and considering the whole country of Portugal as an additional one. Spain is territorially organized into 17 autonomous communities and 2 autonomous cities, which are further divided into a total of 50 provinces. The absence of sampling in certain autonomous communities or provinces was attributable to the limited number or complete absence of farms within those regions. Isolate-level clonal distributions as percentages were reported for each region as: % for a clonal complex = (number of isolates assigned to that complex/total isolates from the region) × 100. To evaluate the percentage of each detected clonal complex within a given autonomous community a generalized linear mixed model (GLMM) was used including a binomial probability distribution and a logit transformation [ln(*μ*/1-μ)] as a link function (Proc Glimmix, SAS). The model included CC as fixed effect.

An interactive mapping tool, StaphyMAP, was also developed using Power BI. It was primarily designed to provide up-to-date epidemiological information on antimicrobial resistance patterns and geographical distribution of *S. aureus* clones. The platform is freely accessible online.^1^

### 2.5 Evaluation of genomic similarity

To infer genetic relatedness among the sequenced strains, we applied hierarchical agglomerative clustering (HAC), by calculating the distance between clusters as the average pairwise distance between elements in each cluster. The clustering was conducted using the *hclust()* function in R with the following parameters: MASH distance [MinHash-based genomic distance (37)] as the distance metric and average linkage (UPGMA) as the clustering method. The *Finds SNP sites* tool (38), version 2.5.1 + galaxy0 in Galaxy Europe, was used to identify single nucleotide polymorphisms (SNPs), and the *SNP distance matrix* tool (39), version 0.8.2 + galaxy0, was used to obtain the number of SNPs between sample pairs.

### 2.6 Antibiotic susceptibility testing

#### 2.6.1 Disk diffusion testing

A total of 353 *Staphylococcus aureus* isolates were selected for antimicrobial susceptibility testing. These isolates represented one strain of each different genotype identified per rabbitry, ensuring representation of the genetic diversity observed across the sample set. Antimicrobial susceptibility was evaluated using the disk diffusion method on Mueller-Hinton agar (MH; Becton-Dickinson, Sparks, MD, USA), following EUCAST guidelines (40, 41) for both methods (Version 13.0) and breakpoints (Version 15.0). For reporting and applicability to the rabbit sector, when EUCAST defined an intermediate category (*I*, susceptible to increased exposure), this category was grouped with resistant (*R*) and reported as “non-susceptible” (*I* + *R*). This was done to (i) maintain comparability with prior rabbit literature (ii) because EUCAST breakpoints use predefined dosing and exposure data for antimicrobial concentrations that is often not achievable in rabbits or other animals, and (iii) because EU and Spanish regulations heavily restrict dose escalation over marketing authorization by the European Medicines Agency (EMA) in food-producing animals (42–44). Accordingly, strains were categorized as either susceptible or non-susceptible. For cross-study comparability, an EUCAST-conformant report including %*S*, %*I*, and %*R* was included (Supplementary File 1). Antimicrobials were selected based on the following criteria: (1) common use in rabbit farming for treatment of *S. aureus* infections, (2) representation of the four categories outlined by the EMA (45), and (3) inclusion of agents classified as critically important for human medicine (46). When specific molecules could not be tested, appropriate control molecules from their respective antimicrobial class were used as replacements. The final antimicrobial panel included: benzylpenicillin (1 U), cefoxitin (30 μg), ceftaroline (5 μg), clindamycin (2 μg), chloramphenicol (30 μg), erythromycin (15 μg), gentamicin (10 μg), kanamycin (30 μg), linezolid (10 μg), norfloxacin (10 μg), tetracycline (30 μg), tigecycline (15 μg), and trimethoprim/sulfamethoxazole (1.25/23.75 μg) (Liofilchem, Teramo, Italy). Strains were classified as multidrug-resistant (MDR) when they exhibited resistance to at least one antimicrobial agent in a minimum of three different antimicrobial classes, following previous reports (47).

A Generalized Linear Mixed Model (GLMM) statistical test with a binomial distribution and a logit link function was used to evaluate resistance frequencies. The model included the interaction between the clonal complex (10 levels) and antimicrobial class (11 levels) as fixed effects. Pairwise comparisons were adjusted using Tukey’s method for multiple comparisons (Proc Glimmix, SAS).

#### 2.6.2 Molecular characterization of methicillin resistance

Additionally, PCR amplification was performed on the 353 isolates described in the previous section to detect the *mec*A and *mec*C genes and further characterize methicillin-resistant *S. aureus* (MRSA) strains. Primers were selected based on previously published protocols for *mec*A (48) and *mec*C (49).

#### 2.6.3 Minimum inhibitory concentration determination

For isolates exhibiting cefoxitin resistance by disk diffusion and/or testing positive for *mec*A or *mec*C, the minimum inhibitory concentration (MIC) was determined using cefoxitin (0.016–256 μg/mL) MIC test strips (Liofilchem, Teramo, Italy), following EUCAST guidelines. Statistical analyses were performed using R version 2023.12.0 + 369. To compare the MIC values among different CCs, the Kruskal–Wallis test was used, as the data did not follow a normal distribution (assessed using Shapiro–Wilk). When significant differences were detected (*p* < 0.05), Dunn’s *post-hoc* test with Bonferroni correction was applied for multiple pairwise comparisons. Adjusted *p*-values were used to assign significance groupings. These analyses were conducted using R packages including *FSA*, *multcompView*, *ggplot2*, *hrbrthemes*, *dplyr*, *clipr*, and *viridis*.

## 3 Results

### 3.1 Strain diversity

From the 783 samples studied, a total of 637 *S. aureus* isolates were identified. After genotyping, 96 different genotypes were described (Supplementary File 2). Most of these genotypes were sporadic, with only 12 genotypes identified in more than 10 isolates, while the remaining 84 were found in fewer than 10. The four most common genotypes (‘A1 II1 *η*’, ‘A1 II1 o’, ‘A1 II1 *λ*,’ and ‘A1 II1 *δ*’) accounted for nearly half of the isolates (48.7%). MLST profiles were obtained for the 243 sequenced isolates, identifying their corresponding CCs and STs. A high level of concordance was observed between the genotypes (assigned from the *coa*-*spa*-*clf*B genotyping) and the MLST profiles (assigned from sequencing) with a 0% error rate for CCs and 10.7% for STs. All genotypes were consistently associated with a single clonal complex, enabling reliable CC assignment. In contrast, some genotypes were linked to multiple sequence types, which accounts for the higher discordance observed at the ST level. Based on this data, CCs and STs were inferred for the full dataset (n = 637), allowing the classification of all isolates. In total, 10 CCs and 35 STs were defined (Table 1; Supplementary Files 2, 3). Isolates for which ST inference was not possible due to equal likelihood of association with multiple STs, were classified as “undefined.”

**Table 1:** Number of identified isolates classified by clonal complex (CC) and sequence type (ST).

CC	ST	*n*
1	1	10
5	146	15
8	407	3
	2,951	1
	7,878*	1
15	15*	3
45	45	1
96	96	27
	2,855	85
	5,001	1
	7,855*	1
	8,008*	8
	8,010*	1
	8,758*	4
	8,759*	5
	8,760*	1
	Undefined^a^	2
121	121	166
	3,764	245
	7,763	8
	7,876*	1
	8,003*	1
	8,009*	1
	8,727*	3
	8,757*	7
	8,763*	3
130	1,945	2
	4,774	8
	7,853*	3
398	398	9
	7,854*	1
	7,877*	1
425	425	1
	8,144*	2
	Undefined^a^	6

*Novel MLST profiles, first described in this study.

a Isolates for which inference was not possible due to equal likelihood of association with multiple categories, were classified as “undefined”.

The dominance of two CCs within the studied population was observed, with CC121 being the most isolated, identified in over two-thirds (*n* = 435; 68.3%) of the positive samples. CC96 followed, with an isolation frequency of 21.2% (*n* = 135). Both CCs accounted for 89.5% of the total studied isolates. Within CC121, the most frequently isolated STs were ST3764 (*n* = 245; 56.3%) and ST121 (*n* = 166; 38.2%). Similarly, ST2855 (*n* = 85; 63%) was the most common within CC96, followed by ST96 (*n* = 27; 20%). The remaining isolates represented 10.5% of the total and were classified into eight different CCs and 31 STs, with six isolates remaining “undefined.”

Among the 285 studied farms, CC121 was isolated from 216 (75.8%), while CC96 was found in 68 (23.9%). At least one of these CCs was identified in 260 of the evaluated farms (91.2%). The remaining CCs were isolated from 46 farms in total (16.1%). More than one positive sample was obtained in 144 farms. A single CC was detected in 106 of them (73.6%), and multiple CCs were identified in the remaining 38 (26.4%). Specifically, two CCs were detected in 35 farms, three in two farms, and four in one farm. Farms where multiple CCs were detected, always included strains belonging to CC121 and/or CC96 (Supplementary File 4).

Regarding the number of isolates per swab, a single CC was identified in most samples (96.2%). However, two CCs were detected in 12 of them. Eleven involved combinations that included CC121 and/or CC96, while the remaining sample contained a combination of CC425 and an undefined isolate.

### 3.2 Geographical distribution

The geographical distribution of CCs was analyzed across 14 autonomous communities (out of 17) and 39 provinces (out of 50) in Spain and five Portuguese districts.

**Figure 1:**
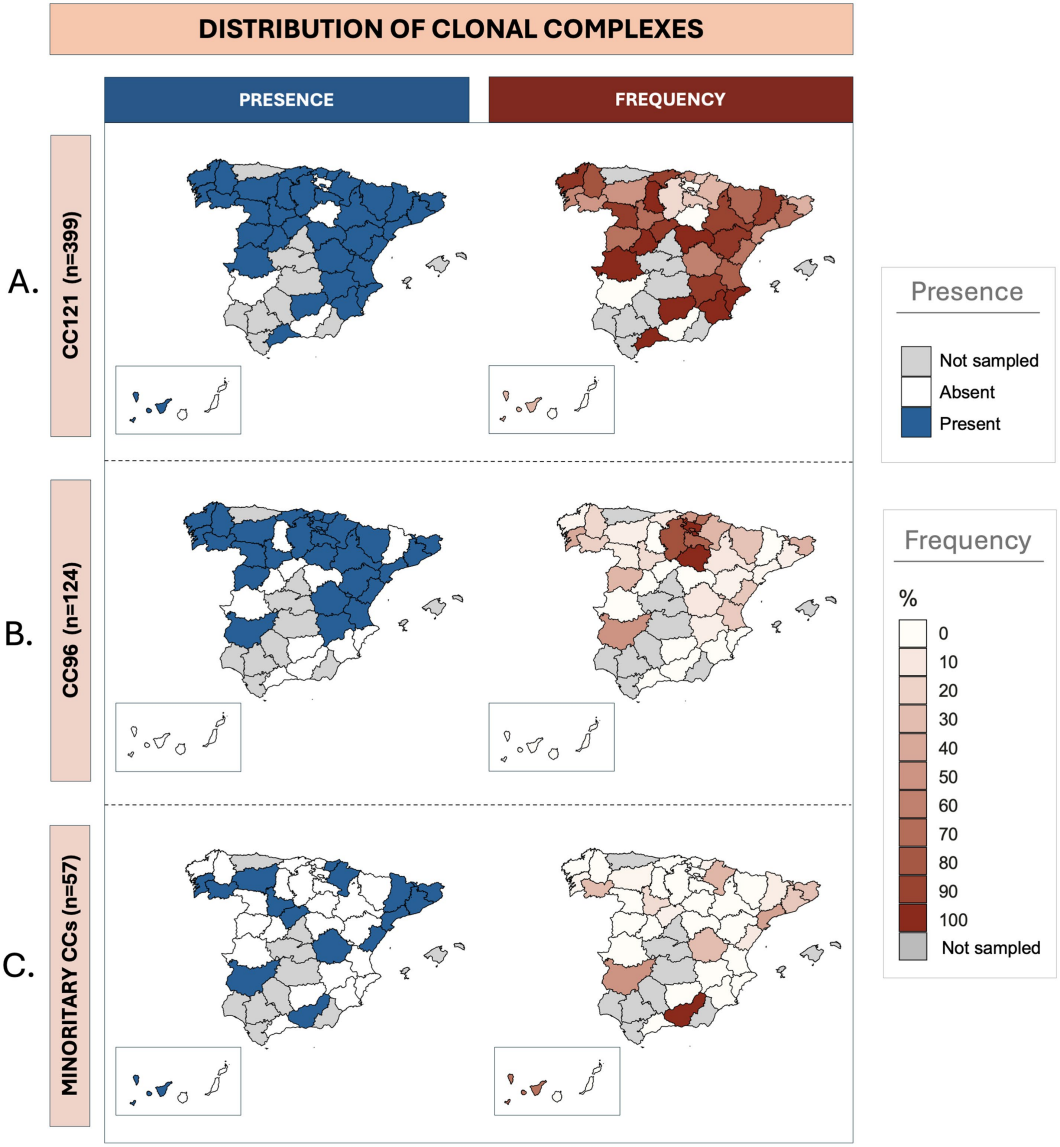
Geographic distribution of *Staphylococcus aureus* clonal complexes isolated from rabbitries across Spanish provinces. For each clonal complex, the maps on the left illustrate the presence (blue) or absence (white), while the ones on the right represent the frequency (red gradient) or absence (white) for each specific territory. Provinces where no samples were obtained are colored in grey. **(A)** Presence and frequency of isolates belonging to CC121. **(B)** Presence and frequency of isolates belonging to CC96. **(C)** Presence and frequency of isolates belonging to the remaining eight sporadic clonal complexes isolated in this study (CC130, CC398, CC5, CC1, CC8, CC15, CC45, and CC425).

As summarized in Table 2, CC121 was detected in all sampled autonomous communities, while CC96 was identified in 12 of them (80%). Meanwhile CC5, CC15, CC130, and CC398 were found in four or three (26.7–20%, respectively); and CC1, CC8, and CC425 were identified in two (13.3%). Clonal complex CC45 was exclusively isolated in *Navarra* (northern region). The two most isolated CCs, CC121 and CC96, showed significantly higher frequencies than others (*p* < 0.05) in seven and two autonomous communities, respectively. Even in regions such as *Cataluña* or *Portugal* (northeast and west, respectively), two of the territories with the highest clonal diversity, these CCs (121 and 96) remained significantly more frequent than the others. To study the impact of these strains on each Spanish province, a relative frequency analysis was conducted considering the number of strains from each CC relative to the total isolates obtained in that specific geographical area (Figure 1 and Supplementary File 5). CC121 stood out as the most widely distributed CC (Figure 1A), detected in 35 out of the 39 sampled provinces (89.7%), with notable presence in *León*, *Valencia*, and *Albacete* (geographically dispersed across the country). CC96 displayed a wide yet more restricted distribution (Figure 1B), being isolated in 27 provinces (69.2%), predominantly in northern (*Pontevedra* and *Gipuzkoa*), and eastern (*Valencia*) provinces of Spain. The remaining CCs exhibited a more localized and sporadic distribution (Figure 1C), isolated in 16 provinces in total (41%). The CC398 and CC5 were the most distributed within this group, found in 7 and 5 provinces, respectively. The rest were detected in 3 or fewer provinces.

**Table 2:** Clonal complex (CC) isolation frequencies (%) classified by autonomous community.

Autonomous communities	CC											*P-*value
	121	96	398	5	Undefined	15	130	1	8	425	45	
Andalucía (*n* = 3)	66.7		33.3									0.468
Aragón (*n* = 41)	87.8^b^	12.2^a^										0.001
Islas Canarias (*n* = 3)	33.3			66.7								0.468
Cantabria (*n* = 11)	90.9^b^	9.1^a^										0.006
Castilla la Mancha (*n* = 43)	83.7^b^	7.0^a^		2.3^a^	2.3^a^	2.3^a^				2.3^a^		0.001
Castilla y León (*n* = 161)	83.2^c^	12.4^b^	2.5^a^	1.2^a^		0.6^a^						0.001
Cataluña (*n* = 87)	62.1^b^	9.2^a^	4.6^a^	11.5^a^				8.0^a^	4.6^a^			0.001
Com. Valenciana (*n* = 75)	70.7^c^	22.7^b^			2.6^a^			4.0^a^				0.001
Extremadura (*n* = 6)	33.3	33.3					33.3					1.000
Galicia (*n* = 107)	67.3^d^	20.6^c^	0.9^a^		1.9^a^		8.4^b^		0.9^a^			0.001
La Rioja (*n* = 8)	25.0	75.0										0.078
Murcia (*n* = 8)	100.0											1.000
Navarra (*n* = 3)	33.3	33.3									33.3	1.000
País Vasco (*n* = 61)	31.1^b^	63.9^c^			1.7^a^					3.3^a^		0.001
Portugal* (*n* = 18)	16.7^a^	61.1^b^	5.6^a^			5.6^a^	11.1^a^					0.002

*Portugal is an independent country but has been included as a territorial unit comparable to a Spanish autonomous community for comparative purposes (see Methods). ^a,b,c,d^ For a given autonomous community, the isolation frequencies of the CCs that do not share a lowercase letter differ significantly (*p* < 0.05).

CC121 achieved a mean frequency of 66% across the sampled territories, whereas the mean frequency for CC96 was 22.2%. The sporadic CCs collectively accounted for the remaining 11.8% of the sample. Analyzing the specific distribution of CC121, in 14 provinces (14/39) its isolation frequency exceeded 90%. This was observed in some highly sampled provinces such as *A Coruña* (northwest) or *Albacete* (eastern Spain), as well as in less intensely sampled provinces like *Segovia*, *Murcia*, or *Lleida* (central and eastern regions). Overall, this CC showed a relative isolation frequency greater than 50% in 28 provinces (28/39). On the other hand, in the case of CC96, despite being isolated in approximately 70% of the sampled territory, its relative frequency exceeded 50% in only six provinces (6/39), showing a smaller impact on the rabbit farms. The territories with higher frequencies were concentrated in the northern areas, particularly in provinces belonging to the autonomous communities of *País Vasco* and *La Rioja* (northern regions). Finally, for the infrequent CCs, lower isolation frequencies were observed. In provinces where more than five samples were obtained, the CCs that achieved above-average frequencies were CC1 in *Barcelona* (12.5%; northeast), CC5 in *Tarragona* (32.1%; northeast), CC8 in *Girona* (21.2%; northeast), and CC130 in *Ourense* (27.3%; northwest).

**Figure 2:**
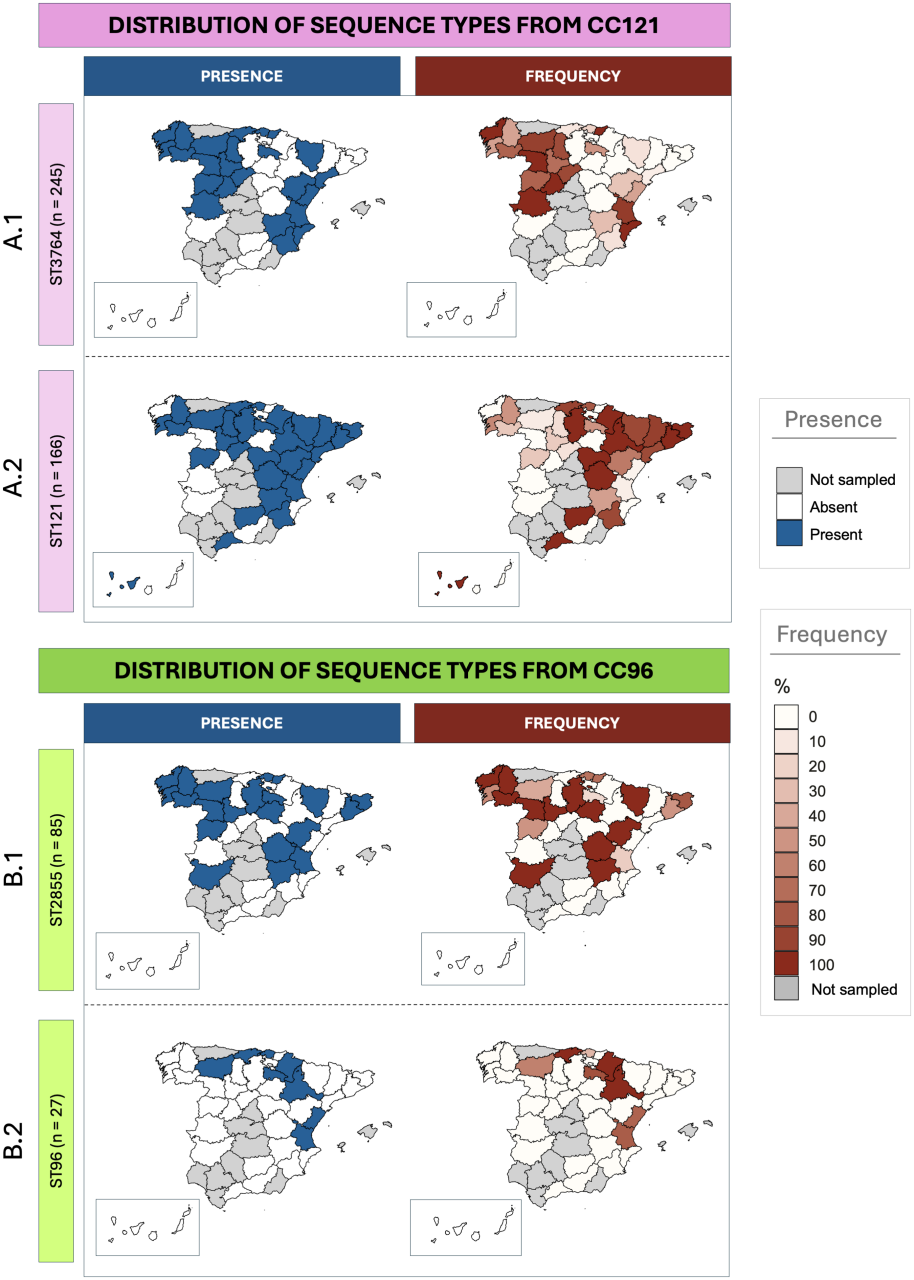
Geographic distribution of *Staphylococcus aureus* sequence types isolated from rabbitries across Spanish provinces. The maps on the left illustrate the presence (blue) or absence (white) of specific isolates, while the ones on the right represent the frequency (red gradient) or absence (white) for each specific territory. Provinces where no samples were obtained are shown in grey. **(A)** Distribution of the two most frequent sequence types within CC121 (**A.1**: ST3764; **A.2**: ST121). **(B)** Distribution of the two most frequent sequence types within CC96 (**B.1**: ST2855; **B.2**: ST96).

The analysis of the distribution of the STs belonging to the most frequent CCs revealed specific distribution patterns (Figure 2). For CC121, while ST3764 was the most frequently isolated, it was detected in 24 provinces (24/39), compared to ST121 which was less isolated but more distributed, detected in 29 provinces (29/39). In Figure 2A, the relative frequencies of both main STs within CC121 is illustrated. ST3764 was predominantly found in the western region of the country, as well as in the region of *Valencia*, in the east. In contrast, ST121 was more frequently isolated in the north-east, with additional sporadic detections in the south-east (certain provinces of *Andalucía* and *Islas Canarias*). Despite these differences, in some provinces such as *La Rioja*, *Lugo*, and *Pontevedra* (located north-west), both STs had similar frequencies. Regarding CC96 (Figure 2B), the most widespread ST was also the most frequently sampled. ST2855 was identified in 21 provinces, whereas ST96 was only isolated in 8. Unlike ST121 and ST3764, no clear distribution pattern was observed for these STs.

All the data was incorporated in an open-access and interactive tool, StaphyMAP, that was developed to support a continuous data update on clonal distribution and prevalence, and to ease the interpretation of the geographical patterns observed. This resource enables the visualization of the spatial distribution of isolates across provinces and autonomous communities, and it also integrates supplementary materials such as images and popular science articles related to rabbit staphylococcosis, allowing users to filter and access relevant information interactively.

### 3.3 Genomic distance approach

To assess the genomic similarity among the 243 sequenced strains, pairwise genomic distances were calculated using the MASH algorithm, which estimates genome similarity through sketching (Supplementary File 6). The observed distances ranged from 0 to 0.023. Some CCs clustered more closely, such as CC121, CC425, and CC130, whereas CC96 showed greater proximity to CC15. Within CC5, one isolate from *Islas Canarias* showed substantial genetic divergence from the other CC5 strains (MASH distance = 0.021), despite being recovered from the same host species. This strain instead clustered with a CC45 genome. Interestingly, a genetic distance of zero was observed among a subset of CC121 strains, some of which were sampled from geographically distant farms with no direct contact or shared personnel.

To further explore strain pairs exhibiting the highest genomic similarity, single nucleotide polymorphisms (SNPs) were analyzed among strain pairs with MASH distances ≤0.005, indicating ≥99.5% genomic similarity and defining genomovars (50). A general trend was observed, with lower SNP counts associated with shorter geographic distances between strains. Strain pairs from the same province exhibited a lower average SNP count (159) compared to the population mean (307.4), which further decreased to 103.5 among strains isolated from the same farm. To examine the most similar isolates in detail, thresholds were further defined using the 10th and 1st percentiles of the SNP distribution (<66 and <26 SNPs, respectively). Notably, at the 10th percentile, most comparisons involved strain pairs from different farms and provinces. Even when the threshold was further reduced to the 1st percentile, 61.5% of the comparisons still involved strain pairs from different provinces, revealing that some of the most genetically similar strains were separated by considerable geographical distances.

Finally, genetic stability within farms was assessed using 38 pairwise SNP comparisons of samples from the same ST and farm, collected at different time points (6–376 days apart). SNP counts ranged from 3 to 1,106: 8 pairs had fewer than 26 SNPs, 12 fell between 26 and 66, and 18 exceeded that range. No consistent relationship was observed between SNP distance and time interval. Notably, several comparisons with lower SNP counts (≤26 SNPs) corresponded to longer time intervals, exceeding 200 days between sampling points. Conversely, multiple comparisons with higher SNP counts (>100 SNPs) had relatively short time intervals, in some cases under 30 days.

### 3.4 Antibiotic susceptibility testing

#### 3.4.1 Disk diffusion testing

Antibiotic susceptibility of the 353 selected isolates, representing one strain per genotype and rabbitry, was assessed calculating the percentage of non-susceptible (*I* + *R*) strains for each antimicrobial class tested (Table 3). Additionally, antimicrobials were classified according to the Antimicrobial Advice *Ad Hoc* Expert Group (AMEG) categorization (45). The highest resistance rates were observed for fluoroquinolones (100%, Category B), followed by tetracyclines (76.8%, Category D) and lincosamides (71.4%, Category C). In contrast, lower resistance levels were found for amphenicols (3.1%, Category C), glycylcyclines (2.5%, Category A), and oxazolidinones (2.5%, Category A). Resistance rates tended to be lower in AMEG categories A and B (high-priority antimicrobials) and higher in categories C and D (more commonly used in veterinary medicine), reflecting usage patterns. However, some exceptions were noted. Moderate resistance was observed for sulfonamides and amphenicols despite their D and C classification, respectively; and high resistance was observed for fluoroquinolones, despite being classified within Category B. Overall, 86.1% of isolates (*n* = 304) were classified as multidrug-resistant (MDR) strains. Notably, all strains showing resistance to antimicrobial classes that generally showed low resistance rates (cephalosporins, sulfonamides, amphenicols, glycylcyclines, and oxazolidinones) also presented MDR profiles.

**Table 3:** Number and percentage of non-susceptible (*I* + *R*) and multidrug-resistant (MDR) strains, grouped by antimicrobial class and AMEG categorization.

Antimicrobial class	AMEG*	Non-susceptible strains	MDR strains
Fluoroquinolones	B	353 (100%)	304 (86.1)
Tetracyclines	D	271 (76, 8%)	266 (91.7)
Lincosamides	C	252 (71, 4%)	251 (97.3)
Macrolides	C	218 (61, 8%)	218 (99.5)
Aminoglycosides	C	205 (58, 1%)	203 (99)
Penicillins	D	131 (37, 1%)	125 (95.4)
Cephalosporins	A	52 (14, 7%)	52 (100)
Sulfonamides	D	48 (13, 6%)	48 (100)
Amphenicols	C	11 (3, 1%)	11 (100)
Glycylcyclines	A	9 (2, 5%)	9 (100)
Oxazolidinones	A	9 (2, 5%)	9 (100)

*Antimicrobial Advice Ad Hoc Expert Group (AMEG) categorization: Category A (Avoid), B (Restrict), C (Caution), and D (Prudence); based on their relevance to human medicine and the risk of resistance development (45).

Analysis of the average resistance to each antimicrobial class across CCs (Table 4) revealed significant differences between groups. Resistance to aminoglycosides was predominantly associated with CC121, showing significantly higher levels than other CCs. For cephalosporins, isolates belonging to CC130 and CC5 showed significantly higher resistance levels. Resistance to lincosamides varied between CCs, with approximately half of them being highly resistant. Macrolide resistance was significantly higher in strains from CC15, CC121, and CC130. The highest resistance to sulfonamides was detected in strains from CC1 and CC96. Resistance to fluoroquinolones, penicillins, and tetracyclines was broadly distributed across CCs, showing no significant differences between them. In contrast, glycylcyclines, amphenicols, and oxazolidinones were widely susceptible.

**Table 4:** Average resistance (%) to antimicrobial classes according to the clonal complex (CC) of *S. aureus* strains.

CC	Antimicrobial class											*P-*value
	Amin	Amph	Cepha	Fluor	Glyc	Linco	Macro	Oxazo	Penici	Sulfo	Tetra	CC
1	33^ab^	0	0^a^	100	0	78^b^	67^ab^	0	11	44^b^	67	0.421
5	44^b^	0	67^c^	94	0	33^a^	33^a^	0	89	0^a^	56	0.147
8	20^a^	0	0^a^	100	0	20^a^	20^a^	0	20	0^a^	40	1.000
15	0^a^	33	0^a^	50	0	100^b^	100^b^	0	100	0^a^	33	1.000
45	0^a^	0	0^a^	50	0	0^a^	0^a^	0	0	0^a^	0	1.000
96	41^bC^	4^A^	8^aA^	94^E^	3^A^	62^aD^	36^aBC^	4^A^	25^B^	45^bC^	75^D^	<0.001
121	61^cC^	2^A^	4^aA^	93^E^	3^A^	77^bD^	75^bD^	2^A^	35^B^	1^aA^	81^D^	<0.001
130	11^aA^	0^A^	94^cC^	100^C^	0^A^	78^bBC^	78^bBC^	0^A^	100^C^	0^aA^	56^B^	0.043
398	55^bB^	9^A^	32^bAB^	91^C^	0^A^	100^bC^	45^aAB^	0^A^	100^C^	9^aA^	100^C^	0.017
425	0^a^	0	0^a^	83	0	0^a^	0^a^	0	0	0^a^	67	1.000
*P-*value class	<0.001	0.710	<0.001	0.105	1.000	0.040	<0.001	1.000	0.219	<0.001	0.204	

^a,b,c^For a given antimicrobial class, the mean values of CCs that do not share a lowercase letter differ significantly (*p* < 0.05). ^A,B,C,D,E^For a given CC, the mean values of antimicrobial classes that do not share an uppercase letter differ significantly (*p* < 0.05).

CC, clonal complex; Amin, aminoglycosides; Amph, amphenicols; Cepha, cephalosporins; Fluor, fluoroquinolones; Glyc, glycylcyclines; Linco, lincosamides; Macro, Macrolides; Oxazo, oxazolidones; Penici, penicillins; Sulfo, sulfonamides; Tetra, tetracyclines.

Within individual CCs, significant differences in resistance patterns were identified in CC96, CC121, CC130, and CC398. These CCs showed marked susceptibility to amphenicols, glycylcyclines, and oxazolidinones. They also displayed significant susceptibility to sulfonamides except for CC96, which was significantly more resistant. Strains from CC96 and CC121 were notably more susceptible to cephalosporins, whereas they were consistently associated with the highest resistance levels to fluoroquinolones, lincosamides and tetracyclines.

With the additional incorporation of antimicrobial resistance data, StaphyMAP can also serve as a decision-support tool for clinical veterinarians, enabling evidence-based treatment strategies during clinical outbreaks.

#### 3.4.2 Molecular characterization of methicillin resistance

Regarding the presence of *mec*A and *mec*C genes, 4.2% of the isolates (*n* = 15) tested positive for *mec*A, and 3.1% (*n* = 11) for *mec*C. Among these, two double-positive isolates (carrying both *mec*A and *mec*C) were identified in CC121 (ST3764) and CC96 (ST5001). In total, 24 isolates (6.8%) were positive for at least one of the methicillin-resistance genes, which were identified across CC130 (*n* = 9; 100%), CC5 (*n* = 8; 88.9%), CC398 (*n* = 4; 36.4%), CC121 (*n* = 2; 0.9%), and CC96 (*n* = 1; 1.1%).

Discrepancies between genotypic and phenotypic resistances were observed. Two *mec*-positive isolates (one double-positive and one *mec*A-positive) exhibited phenotypic sensibility to cefoxitin by disc diffusion, whereas 24 isolates that were PCR-negative for both *mec*A and *mec*C genes displayed resistance.

#### 3.4.3 Cefoxitin minimum inhibitory concentration determination

**Figure 3:**
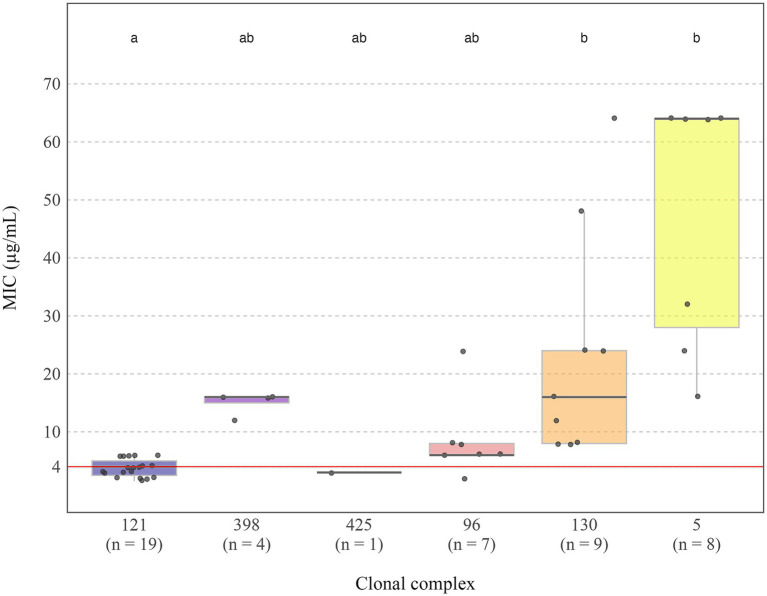
Distribution of MIC values (μg/mL) by clonal complex in clinical *S. aureus* rabbit isolates. The red dashed line at MIC = 4 μg/mL represents the EUCAST clinical breakpoint for resistance classification. Letters above each group indicate statistically significant differences in MIC distributions between clonal complexes (*p* < 0.05): groups not sharing the same letter differ significantly. Sample sizes for each group are shown in parentheses below the *x*-axis labels. One outgroup (CC5, MIC >256 μg/mL) was excluded for visual clarity.

To clarify genotypic-phenotypic discrepancies and to quantify cefoxitin resistance, MIC testing was performed on all discordant isolates, as well as all isolates classified as cefoxitin resistant by disc diffusion. A total of 48 isolates were studied. MIC values ranged from 1.5 μg/mL to 64 μg/mL (Figure 3), except for one isolate, belonging to CC5 (ST146) and carrying the *mec*A gene, which exhibited a MIC value above the upper limit of the test strip range (256 μg/mL).

Resistance was detected in 100% of CC130 isolates (9/9), 89% of CC5 (8/9), 36% of CC398 (4/11), 7% of CC96 (6/91), and 2% of CC121 (5/212). All isolates characterized as MRSA, were MDR. A comparative analysis of MIC values across CCs revealed statistically significant differences. CC121 showed the lowest and most consistent MIC values (1–6 μg/mL), with the majority below the EUCAST resistance breakpoint (MIC > 4 μg/mL). In contrast, CC130 and CC5 presented significantly higher MIC values (8–64 μg/mL and 16–256 μg/mL, respectively), all exceeding the threshold. CC5 also displayed the widest MIC range.

Despite cefoxitin MIC testing, some discrepancies between phenotype and genotype persisted. The two isolates carrying resistance genes that initially tested susceptible by disc diffusion remained susceptible by MIC testing. Among the 24 isolates that showed cefoxitin resistance through disk diffusion but lacked *mec* genes, MIC testing reclassified 14 of them as susceptible, while 10 remained resistant despite the absence of an identified resistance gene.

## 4 Discussion

The analysis of the genetic diversity and antimicrobial resistance of *Staphylococcus aureus* in rabbit farming is essential to understand transmission dynamics and develop effective control strategies. In this study, a broad and geographically diverse sampling effort was conducted across the Iberian Peninsula, providing a comprehensive overview of the population structure, clonal distribution and antimicrobial resistance profiles of *S. aureus* in commercial rabbit production.

### 4.1 Strain diversity and distribution

Our results reveal considerable genetic diversity, with 96 different genotypic combinations and 35 sequence types identified, including five previously undescribed coagulase types, 10 new protein A types, and three novel clumping factor B types, expanding upon previous studies (7, 18, 21). However, despite this high genotypic diversity, only a few clones showed widespread distribution: four genotypes (A1 II1 *η*, A1 II1 o, A1 II1 *λ*, A1 II1 *δ*) accounted for nearly half (48.7%) of all isolates. Among these, ‘A1 II1 η’ and ‘A1 II1 δ’ maintaining their predominance as previously reported in Spain (7), while ‘A1 II1 o’ and ‘A1 II1 λ’ were newly identified here.

MLST analysis confirmed the dominance of two CCs, CC121 and CC96, which together represented 89.5% of isolates, consistent with prior reports from Europe and China (8–10, 51). CC121 was the most frequently isolated, with a 13.1% increase in detection compared to earlier studies (7), accompanied by a corresponding decrease in CC96 and minor CCs. Within CC121, ST3764 increased notably (+19.8%), while ST121 decreased (−8.6%), indicating a population shift favoring ST3764. Although less frequent, CC96 remained epidemiologically relevant with a 21.2% isolation frequency. The dominance of these CCs suggests selective advantages, supported by previous studies showing CC121’s higher virulence and abscess-forming capacity in rabbits compared to CC96 (8). Additionally, farms with multiple CCs consistently included CC121 and/or CC96, reinforcing their central epidemiological role. Genomic distance analysis indicated that CC121 strains were genetically closer to CC130 and CC425, while CC96 clustered with CC15, suggesting potentially shared genomic traits such as virulence factors, resistance genes, or mobile genetic elements (52). These genomic similarities could potentially contribute to gene exchange and promote increased pathogenicity or antimicrobial resistance (53).

The isolation frequencies were consistent with the clonal distribution throughout the Iberian Peninsula, with CC121 widely distributed and CC96 also present, though less frequently. The broad geographic presence of both lineages, even in provinces with higher sampling densities, supports their adaptation to rabbits and efficient transmission dynamics. In contrast, other CCs appeared sporadically, likely reflecting limited adaptation or recent introductions. Regional variation within CC121 and CC96 was observed. For instance, ST3764 predominated in western regions and *Comunidad Valenciana* (east), whereas ST121 was more common in the east. These patterns, in line with previous findings (7), likely reflect local transmission networks, trade routes, or different environmental and management conditions. Within CC96, although no clear pattern was identified for ST2855, it was detected at relatively high frequency across several autonomous communities distributed throughout the entire sampled territory. This differs from the results offered by previous work (7), where this ST was identified predominantly in northern regions. This divergence likely reflects the increased sampling depth and geographical coverage of the current study, which may capture a more accurate picture of the epidemiological distribution of ST2855 in commercial rabbit farming, as well as the possibility that these strains have expanded over time, becoming dominant.

Less frequent lineages were detected in 26.4% of farms, often at low frequencies (<3% each), including 17 novel profiles and one (ST15) previously found in other animals and humans but not in rabbits (54). These may represent sporadic introductions via animal transport or contact with humans or wildlife, especially in farms with poor biosecurity measures. Their low frequencies may indicate limited adaptation to the rabbit host or recent emergence (55), as has been the case for ST3764, which was first isolated in this population in 2014 (7) and now accounts for 56.3% of the isolated strains. While *S. aureus* is typically host-specialist (54), it also exhibits remarkable transmission and adaptation capabilities across hosts (56). Host-jump events involving humans have been documented for several CCs identified here, such as CC398 in pigs (57), CC130 in hedgehogs (58), CC1 in horses (59), CC5 in poultry (60), or CC121 in rabbits (61). These CCs have also been isolated from Iberian wildlife: small mammals (62, 63), birds (63, 64), wild rabbits (65), and from larger species such as wild boars and red deer (63, 66). While the occasional detection of some of these lineages in domestic rabbits suggests possible transmission from wildlife, data suggests these strains have not yet become established in rabbit farms. Nevertheless, their capacity to cause infections underscores the potential role of wildlife as a reservoir for occasional host-jump events, particularly when biosecurity measures are insufficient.

SNP analysis revealed minimal variation among some isolates from the same farm over long time periods, suggesting genomic stability and potential long-term persistence of specific clones, once established. These findings, although based on a limited number of comparisons derived from a random sampling strategy, point to the possibility of stable colonization dynamics under current production conditions. This stability may be facilitated by intensive farming practices, including large breeding populations (mean 800 does), short reproductive cycles (42-day kindling intervals), and the continuous introduction of susceptible animals (67), which together promote transmission and reinfection. These findings, though exploratory, underscore the potential for strains to persist in farm environments over time despite sanitary measures, and highlight the importance of targeted longitudinal studies to better characterize this persistence from both an epidemiological and genomic perspective. Lastly, although genetic similarity generally correlated with geographic proximity, closely related CC121 strains (<26 SNPs) were found in geographically distant farms, suggesting potential clonal dissemination. This could be related to productive practices such as animal exchange between farms, a shared origin of breeding stock, or the use of artificial insemination. However, further studies are required to confirm the source of this dissemination. Furthermore, strain stability has been linked to genomic traits that promote adhesion, immune evasion, and environmental stress resistance (68), reinforcing the idea that dominant, stable strains, such as CC121, might possess genomic adaptations, favoring persistence in rabbitries.

Altogether, our data indicates that CC121 and CC96 continue to dominate in the rabbit population and have remained stable since the earliest reports. However, previously undescribed CCs in rabbitries were also identified with the sporadic, but concerning, detection of lineages from external sources such as CC1, CC15, or CC45.

### 4.2 Antimicrobial resistance

Antimicrobial resistance patterns in *S. aureus* isolated from rabbits exhibit marked variability across studies, highlighting the dynamic nature of resistance in this host. Several European research works have reported high resistance rates to macrolides, lincosamides, and *β*-lactams (22, 69), whereas others, conducted in different geographical contexts, described broader susceptibility across most antimicrobial families (10, 70). These discrepancies likely result from differences in local antibiotic usage patterns, therapeutic strategies, or regulatory frameworks affecting antibiotic prescription and resistance selection. In Spain, previous studies have reported high resistance rates to cephalosporins (23), fluoroquinolones and *β*-lactams in farmed rabbits (71), and notably, to *β*-lactams, tetracyclines, and fluoroquinolones in wild rabbits (65). Our findings align with these patterns, showing high resistance rates to tetracyclines and fluoroquinolones, frequently administered in rabbit production, thus supporting the link between antimicrobial use and resistance development. However, despite widespread use, global resistance to penicillins and cephalosporins remained below 40%.

Resistance profiles varied significantly among CCs, indicating a strong influence of genetic background. For instance, CC130 isolates showed high cephalosporin resistance, while CC398 was notably resistant to lincosamides. In contrast, resistance to fluoroquinolones, penicillins, and tetracyclines was widespread across CCs, without clear lineage specificity. Although sulfonamide resistance was generally low, higher levels appeared in CC1 and CC96. In *S. aureus*, resistance determinants can also contribute to ecological success and persistence in the environment. For example, mutations in the quinolone resistance-determining region (QRDR) that enhance fluoroquinolone survival have been linked to clonal success under intermittent drug pressure (72, 73). Moreover, multidrug efflux pumps such as *nor*A/B, *mep*A or *tet*38, can act as persistence factors through antimicrobial tolerance and promoting biofilm formation (74–77). Together, these mutations and efflux mechanisms, among others, could provide pathways for the long-term stability CC96 and CC121 strains.

To align with EUCAST recommendations, we additionally re-analysed susceptibility by separating the intermediate category from resistant (Supplementary File 1; see Methods). This analysis confirmed our overall results, but some discrepancies were noted: average fluoroquinolone resistance varied, becoming significant between CCs and higher for CC1, CC5, CC8, and CC130; and sulfonamide resistance in CC1 strains decreased, leaving CC96 strains significantly more resistant than the rest. This analysis confirms the robustness of the main conclusions of this work but adjusts the magnitude and significance by CC, which is important to consider for comparative studies.

MDR strains were highly frequent (86.1%), exceeding rates reported in previous studies (15, 78) and raising concerns about antimicrobial resistance expansion in rabbit farming. The detection of MDR isolates, particularly in lineages with zoonotic potential, such as CC398, highlights their role as potential reservoirs of clinically relevant resistance determinants.

In this context, the EMA classified antibiotics based on their importance in human medicine and the need to limit their use in veterinary contexts (45), in line with the World Health Organization’s (WHO) list of medically important antimicrobials (46). Consistent with this, higher resistance levels were detected for antimicrobials in categories C and D, commonly used in rabbitries, such as tetracyclines and macrolides. In contrast, amphenicols (Category C) displayed low resistance, likely due to their limited use given their toxicity in rabbits, reinforcing the connection between exposure and resistance selection pressure. Meanwhile, Category A antimicrobials, banned or severely restricted in veterinary medicine due to their critical role in human healthcare, remained largely effective. However, low but concerning resistance levels for glycylcyclines and oxazolidinones, both classified as “highest priority critically important antimicrobials” by the WHO, was detected, particularly in CC121 and CC96 isolates. Similarly, cephalosporins (Category A) also showed moderate resistance in CC398, CC5, and especially high in CC130. These findings underscore the urgent need for antimicrobial resistance surveillance focusing on critically important drugs, essential to human medicine.

MRSA was detected in 9.1% of the 353 tested strains. While *mec* genes were identified in CC121 and CC96, the most frequently isolated CCs in this study, they were not the main contributors to methicillin resistance. Instead, *mec*-positive isolates were primarily associated with CC130 (69.2%), CC5 (53.3%), and CC398 (36.4%), lineages with known multihost potential and frequent human infection associations (54). Their presence suggests possible interspecies transmission and zoonotic risks, with potential environmental dissemination via livestock waste.

Discrepancies between phenotypic and genotypic resistance to cefoxitin suggest alternative methicillin resistance mechanisms or detection limitations. Although MIC testing clarified some cases, 10 isolates were phenotypically resistant yet lacked *mec*A or *mec*C, implicating other determinants such as *mec*B, *mec*D, *mec*E, or altered regulatory pathways (79). Conversely, the detection of *mec* genes in phenotypically susceptible isolates may indicate low gene expression or mutations impairing gene functionality. These findings emphasize the importance of combining phenotypic and molecular diagnostics for accurate methicillin resistance detection.

### 4.3 StaphyMAP

Finally, the StaphyMAP tool represents a valuable resource for the control of *S. aureus* in commercial rabbit farming. By providing a visual and accessible overview of the geographical distribution of clonal lineages, it can support targeted sanitary interventions at the farm level, aid in the prevention and control of outbreaks, and contribute to the surveillance of strains with potential public health relevance. Furthermore, by incorporating antimicrobial resistance data, the tool facilitates evidence-based therapeutic decisions, which may contribute to a reduction in overall antibiotic usage and, consequently, to limit the development and spread of antimicrobial resistance.

## 5 Conclusion

In conclusion, this study provides a comprehensive overview of the genetic structure, dissemination patterns, and antimicrobial resistance profiles of *S. aureus* in commercial rabbit farming. It highlights the notable genetic diversity of *S. aureus*, previously overlooked, with the incorporation of newly identified and highly frequent genotypes. However, strain diversity has remained largely stable over time, dominated by a few well-adapted clones that appear to possess a clear evolutionary advantage in this environment. Their predominance, along with their broad geographic distribution and genomic stability, suggests a high capacity for transmission and long-term persistence. The detection of minor or uncommon lineages points to sporadic introductions, likely linked to commercial practices, human contact, or wildlife reservoirs. Although these clones have not yet become established in farms, their pathogenic and multi-host potential underscores the need to reinforce biosecurity and hygiene measures. The high percentages of antimicrobial resistance and multidrug-resistant strains with zoonotic potential reinforces the importance of ongoing monitoring and control within a One Health framework, especially concerning given the detection of strains resistant to critically important antimicrobials for human medicine. The implementation of sustained and proactive surveillance systems is essential to support the development of targeted treatment strategies and outbreak control measures, ensuring effective management of *S. aureus* in rabbit farming. This effort can be further backed by tools such as StaphyMAP, developed to facilitate the interpretation and application of genomic surveillance data in rabbit farming and potentially in other livestock production systems.

## Data Availability

The sequencing data presented in this study can be found at the NCBI Sequence Read Archive (SRA), under the BioProject number PRJNA1297310.

## Footnotes

^1^ https://app.powerbi.com/reportEmbed?reportId=9fd9ca8a-3147-4d91-a120-328fec9858ed&autoAuth=true&ctid=34330eef-31be-4cd1-925e-a790ac722d30
