# Supplementary material for: Staphylococcus aureus characterization in commercial rabbit farms reveals high genetic diversity and widespread antimicrobial resistance
Source: Front Vet Sci. 2025 Oct 30;12:1673809. doi: 10.3389/fvets.2025.1673809 (PMC12613231; doi:10.3389/fvets.2025.1673809)
Supplement: Supplementary file 1 [file Data_Sheet_1.pdf]

Additional File 1. EUCAST-conformant characterization of *S. aureus* strains isolated from rabbitries in Spain and Portugal.

Results in this additional file confirm that, for our study sample, both approaches (classifying intermediate category together with resistant as presented in the research work, or treating them as separate categories as recommended by EUCAST) led to the same qualitative results, although with slight quantitative discrepancies that did not affect the depicted structure of antimicrobial resistance in the *S. aureus* population in our study.

Resistance classified by clonal complex varied significantly for fluoroquinolones when comparing resistance between clonal complexes, and for sulfonamides' average in CC1.

Table AF1.1. Percentage of resistant, intermediate (susceptible, increased exposure) and susceptible strains classified by antimicrobial class.

**Antimicrobial class**	**Categorization EUCAST-conformant**		
	**Resistant**	**Intermediate**	**Susceptible**
Fluoroquinolones	306 (86,7%)	47 (13,3%)	0%
Tetracyclines	271 (76,8%)	-	82 (23,2%)
Lincosamides	252 (71,4%)	-	101 (28,6%)
Macrolides	218 (61,8%)	-	135 (38,2%)
Aminoglycosides	205 (58,1%)	-	148 (41,9%)
Penicillins	131 (37,1%)	-	222 (62,9%)
Cephalosporins	42 (11,9%)	10 (2,8%)	301 (85,3%)
Sulfonamides	37 (10,5%)	11 (3,1%)	305 (86,4%)
Amphenicols	11 (3,1%)	-	342 (96,9%)
Glycylcyclines	9 (2,5%)	-	344 (97,5%)
Oxazolidinones	9 (2,5%)	-	344 (97,5%)

"–" indicates that EUCAST does not define an intermediate category for antimicrobials in this class.

Table AF1.2. Average resistance (%) to antimicrobial classes according to the clonal complex (CC) of *S. aureus* strains.

CC	Antimicrobial class											*p-value*
	**Amin**	**Amph**	**Cepha**	**Fluor**	**Glyc**	**Linco**	**Macro**	**Oxa**	**Penici**	**Sulfo**	**Tetra**	CC
1	33^{ab}	0	0^{a}	78^{bc}	0	78^{b}	67^{ab}	0	11	0^{a}	67	0.181
5	44^{b}	0	44^{bc}	89^{c}	0	33^{a}	33^{a}	0	89	0^{a}	56	0.177
8	20^{a}	0	0^{a}	90^{c}	0	20^{a}	20^{a}	0	20	0^{a}	40	0.482
15	0^{a}	33	0^{a}	0^{a}	0	100^{b}	100^{b}	0	100	0^{a}	33	1.000
45	0^{a}	0	0^{a}	0^{a}	0	0^{a}	0^{a}	0	0	0^{a}	0	1.000
96	41^{bC}	4^{A}	4^{aA}	63^{aD}	3^{A}	62^{aD}	36^{aBC}	4^{A}	25^{B}	38^{bBC}	75^{E}	<0.001
121	61^{cC}	2^{A}	3^{aA}	80^{bD}	3^{A}	77^{bD}	75^{bD}	2^{A}	35^{B}	0^{aA}	81^{D}	<0.001
130	11^{aA}	0^{A}	61^{cB}	94^{cC}	0^{A}	78^{bBC}	78^{bBC}	0^{A}	100^{C}	0^{aA}	56^{B}	0.042
398	55^{bB}	9^{A}	18^{abA}	68^{abB}	0^{A}	100^{b}	45^{aAB}	0^{A}	100^{C}	9^{aA}	100^{C}	0.044
425	0^{a}	0	0^{a}	67^{ab}	0	0^{a}	0^{a}	0	0	0^{a}	67	1.000
p-value class	<0.001	0.710	<0.001	0.002	1.00	0.040	<0.001	1.00	0.219	0.003	0.204	

^{a,b,c} : For a given antimicrobial class, the mean values of CCs that do not share a lowercase letter differ significantly ($p < 0.05$). ^{A,B,C,D,E} : For a given CC, the mean values of antimicrobial classes that do not share an uppercase letter differ significantly ($p < 0.05$).

CC: Clonal Complex; Amin: Aminoglycosides; Amph: amphenicols; Cepha: Cephalosporins; Fluor: Fluoroquinolones; Glyc: Glycylcyclines; Linco: Lincosamides; Macro: Macrolides; Oxa: Oxazolidones; Penici: Penicillins; Sulfo: Sulfonamides; Tetra: Tetracyclines.
