# Supplementary material for: Staphylococcus aureus characterization in commercial rabbit farms reveals high genetic diversity and widespread antimicrobial resistance
Source: Front Vet Sci. 2025 Oct 30;12:1673809. doi: 10.3389/fvets.2025.1673809 (PMC12613231; doi:10.3389/fvets.2025.1673809)
Supplement: Supplementary file 2 [file Data_Sheet_2.pdf]

Additional File 2. Genotype frequencies across the 637 samples and 285 farms included in the study, including their inferred clonal complexes (CC) and sequence types (ST). Isolates for which inference was not possible due to equal likelihood of association with multiple categories, were classified as "undefined".

GENOTYPE	n Samples	% Samples	n Farms	% Farms	Observed CC	nST	1st assigned ST	2nd assigned ST	3rd assigned ST	4th assigned ST	5th assigned ST
A1 II1 η	137	21.5	65	22.8	121	2	3764	121	-	-	-
A1 II1 ο	63	9.89	40	14.0	121	5	121	8009	3764	7763	8763
A1 II1 λ	57	8.94	33	11.5	121	4	121	8003	8757	8727	-
A1 II1 δ	53	8.32	38	13.3	121	2	121	121	-	-	-
B1 IV2 β	30	4.70	20	7.01	96	5	2855	2855	5001	96	8008
A1 II1 κ	27	4.23	21	7.36	121	3	3764	121	8727	-	-
B1 IV2 α	21	3.29	15	5.26	96	3	2855	96	8008	-	-
B1 IV1 α	19	2.98	11	3.85	96	2	2855	8758	-	-	-
B1 IV1 β	17	2.67	9	3.15	96	2	2855	8759	-	-	-
A1 III6 δ	15	2.35	11	3.85	121	1	121	-	-	-	-
A1 III6 ο	13	2.04	8	2.80	121	2	3764	121	-	-	-
A1 III6 λ	13	2.04	8	2.80	121	1	121	-	-	-	-
B3 IV2 θ	9	1.41	5	1.75	5	1	146	-	-	-	-
A1 II1 ε	7	1.09	6	2.10	121	2	3764	121	-	-	-
A1 III6 η	7	1.09	4	1.40	121	1	3764	-	-	-	-
B1 IV2 γ	7	1.09	4	1.40	96	3	96	Undefined	2855	-	-
B4 IV6 κ	7	1.09	3	1.05	130	2	4774	7853	-	-	-
A1 II1 θ	6	0.94	5	1.75	121	1	3764	-	-	-	-
A1 III1 δ	6	0.94	3	1.05	121	1	121	-	-	-	-
A1 II1 ζ	5	0.78	3	1.05	121	2	121	3764	-	-	-
A1 II1 μ	4	0.62	2	0.70	121	2	7876	121	-	-	-
B1 I1 α	4	0.62	2	0.70	96	1	96	-	-	-	-
B1 I7 α	4	0.62	4	1.40	96	2	96	8008	-	-	-
B1 IV2 κ	4	0.62	2	0.70	96	1	96	-	-	-	-
A1 II1 α	3	0.47	3	1.05	121	1	3764	-	-	-	-
A1 III1 κ	3	0.47	2	0.70	121	2	121	3764	-	-	-
D4 V1 δ	3	0.47	2	0.70	1	1	1	-	-	-	-

GENOTYPE	n Samples	% Samples	n Farms	% Farms	Observed CC	nST	1st assigned ST	2nd assigned ST	3rd assigned ST	4th assigned ST	5th assigned ST
F7 I6 ζ	3	0.47	3	1.05	398	1	398	-	-	-	-
A II1 ο	2	0.31	1	0.35	Undefined	1	Undefined	-	-	-	-
A1 III1 η	2	0.31	2	0.70	121	1	3764	-	-	-	-
A1 III6 κ	2	0.31	1	0.35	121	1	3764	-	-	-	-
B1 I1 β	2	0.31	1	0.35	96	1	2855	-	-	-	-
B1 I11 β	2	0.31	1	0.35	96	1	8008	-	-	-	-
B1 II6 β	2	0.31	1	0.35	96	1	2855	-	-	-	-
B1 IV11 α	2	0.31	2	0.70	96	2	7855	2855	-	-	-
B1 IV2 δ	2	0.31	1	0.35	96	1	2855	-	-	-	-
B1 IV2 λ	2	0.31	2	0.70	96	1	96	-	-	-	-
B1 IV3 β	2	0.31	1	0.35	96	1	2855	-	-	-	-
B3 II6 γ	2	0.31	1	0.35	5	1	146	-	-	-	-
B3 IV2 ε	2	0.31	2	0.70	5	1	146	-	-	-	-
B4 II1 θ	2	0.31	1	0.35	130	1	4774	-	-	-	-
D1 IV2 β	2	0.31	1	0.35	8	2	7878	407	-	-	-
D2 IV2 α	2	0.31	2	0.70	96	1	2855	-	-	-	-
D4 IV2 γ	2	0.31	1	0.35	1	1	1	-	-	-	-
D4 V1 γ	2	0.31	1	0.35	1	1	1	-	-	-	-
D4 V1 θ	2	0.31	2	0.70	1	1	1	-	-	-	-
D5 IV1 β	2	0.31	2	0.70	15	1	15	-	-	-	-
F2 IV9 α	2	0.31	1	0.35	425	1	8144	-	-	-	-
F7 I6 κ	2	0.31	2	0.70	398	1	398	-	-	-	-
F7 I6 μ	2	0.31	1	0.35	Undefined	1	Undefined	-	-	-	-
A1 III1 ο	1	0.15	1	0.35	121	1	3764	-	-	-	-
A1 III1 λ	1	0.15	1	0.35	121	1	121	-	-	-	-
A1 III6 ν	1	0.15	1	0.35	121	1	3764	-	-	-	-
A1 III6 α	1	0.15	1	0.35	121	1	121	-	-	-	-
A1 III6 ε	1	0.15	1	0.35	121	1	121	-	-	-	-
A1 VII1 δ	1	0.15	1	0.35	121	1	121	-	-	-	-
A3 II1 κ	1	0.15	1	0.35	121	1	7763	-	-	-	-

GENOTYPE	n Samples	% Samples	n Farms	% Farms	Observed CC	nST	1st assigned ST	2nd assigned ST	3rd assigned ST	4th assigned ST	5th assigned ST
A4 II1 δ	1	0.15	1	0.35	121	1	121	-	-	-	-
B1 - -	1	0.15	1	0.35	Undefined	1	Undefined	-	-	-	-
B1 I7 β	1	0.15	1	0.35	96	1	2855	-	-	-	-
B1 II1 ο	1	0.15	1	0.35	121	1	7763	-	-	-	-
B1 IV1 κ	1	0.15	1	0.35	96	1	96	-	-	-	-
B1 IV1 γ	1	0.15	1	0.35	96	1	2855	-	-	-	-
B1 IV1 δ	1	0.15	1	0.35	96	1	2855	-	-	-	-
B1 IV10 β	1	0.15	1	0.35	96	1	96	-	-	-	-
B1 IV11 β	1	0.15	1	0.35	96	1	8760	-	-	-	-
B1 IV11 γ	1	0.15	1	0.35	96	1	96	-	-	-	-
B1 IV11 η	1	0.15	1	0.35	96	1	96	-	-	-	-
B1 IV11 κ	1	0.15	1	0.35	96	1	96	-	-	-	-
B1 IV2 i	1	0.15	1	0.35	96	1	96	-	-	-	-
B1 IV2 θ	1	0.15	1	0.35	96	1	8010	-	-	-	-
B1 IV3 α	1	0.15	1	0.35	96	1	96	-	-	-	-
B2 I1 ζ	1	0.15	1	0.35	45	1	45	-	-	-	-
B2 IV2 γ	1	0.15	1	0.35	5	1	146	-	-	-	-
B3 IV2 β	1	0.15	1	0.35	5	1	146	-	-	-	-
B4 I11 γ	1	0.15	1	0.35	130	1	1945	-	-	-	-
B4 III6 κ	1	0.15	1	0.35	130	1	4774	-	-	-	-
B4 IV1 η	1	0.15	1	0.35	130	1	4774	-	-	-	-
B4 IV12 γ	1	0.15	1	0.35	130	1	1945	-	-	-	-
C II1 η	1	0.15	1	0.35	Undefined	1	Undefined	-	-	-	-
C4 IV2 γ	1	0.15	1	0.35	8	1	2951	-	-	-	-
D1 IV1 β	1	0.15	1	0.35	8	1	407	-	-	-	-
D1 IV2 i	1	0.15	1	0.35	8	1	407	-	-	-	-
D2 II1 δ	1	0.15	1	0.35	121	1	121	-	-	-	-
D4 V1 β	1	0.15	1	0.35	1	1	1	-	-	-	-
D5 IV2 γ	1	0.15	1	0.35	15	1	15	-	-	-	-
D8 IV11 α	1	0.15	1	0.35	96	1	2855	-	-	-	-

GENOTYPE	n Samples	% Samples	n Farms	% Farms	Observed CC	nST	1st assigned ST	2nd assigned ST	3rd assigned ST	4th assigned ST	5th assigned ST
F1 II1 δ	1	0.15	1	0.35	121	1	121	-	-	-	-
F2 I12 α	1	0.15	1	0.35	425	1	425	-	-	-	-
F3 II1 δ	1	0.15	1	0.35	121	1	121	-	-	-	-
F7 I6 o	1	0.15	1	0.35	398	1	398	-	-	-	-
F7 I6 α	1	0.15	1	0.35	398	1	398	-	-	-	-
F7 I6 β	1	0.15	1	0.35	398	1	398	-	-	-	-
F7 I6 η	1	0.15	1	0.35	398	1	398	-	-	-	-
F7 IV4 δ	1	0.15	1	0.35	398	1	7854	-	-	-	-
F8 I6 δ	1	0.15	1	0.35	398	1	7877	-	-	-	-
