# Supplementary material for: Staphylococcus aureus characterization in commercial rabbit farms reveals high genetic diversity and widespread antimicrobial resistance
Source: Front Vet Sci. 2025 Oct 30;12:1673809. doi: 10.3389/fvets.2025.1673809 (PMC12613231; doi:10.3389/fvets.2025.1673809)
Supplement: Supplementary file 3 [file Data_Sheet_3.pdf]

Additional File 3. Comparison of expected and observed sequence types (STs) and clonal complexes (CCs) in the 243 sequenced isolates which were used as the guide for inference.

GENOTYPE	EXPECTED ST	n Observed STs	1st Observed ST	2nd Observed ST	3rd Observed ST	4th Observed ST	EXPECTED CC	n Observed CCs	Observed CC
A1 II1 o	3764	4	3764 (n= 24)	7763 (n= 3)	121 (n= 1)	8009 (n= 1)	121	1	121 (n= 29)
A1 II1 α	3764	1	3764 (n= 1)	-	-	-	121	1	121 (n= 1)
A1 II1 β	121	1	121 (n= 1)	-	-	-	121	1	121 (n= 1)
A1 II1 δ	121	1	121 (n= 19)	-	-	-	121	1	121 (n= 19)
A1 II1 ε	121	2	121 (n= 3)	3764 (n= 2)	-	-	121	1	121 (n= 5)
A1 II1 ζ	121	2	121 (n= 3)	3764 (n= 1)	-	-	121	1	121 (n= 4)
A1 II1 η	3764	2	3764 (n= 25)	121 (n= 2)	-	-	121	1	121 (n= 27)
A1 II1 θ	3764	1	3764 (n= 5)	-	-	-	121	1	121 (n= 5)
A1 II1 κ	3764	3	3764 (n= 7)	121 (n= 3)	8727 (n= 1)	-	121	1	121 (n= 11)
A1 II1 λ	121	3	121 (n= 19)	8727 (n= 2)	8003 (n= 1)	-	121	1	121 (n= 22)
A1 II1 μ	121	2	121 (n= 2)	7876 (n= 1)	-	-	121	1	121 (n= 3)
A1 III1 o	3764	1	3764 (n= 1)	-	-	-	121	1	121 (n= 1)
A1 III1 δ	121	1	121 (n= 3)	-	-	-	121	1	121 (n= 3)
A1 III1 η	3764	1	3764 (n= 1)	-	-	-	121	1	121 (n= 1)
A1 III1 κ	121	2	121 (n= 1)	3764 (n= 1)	-	-	121	1	121 (n= 2)
A1 III1 λ	121	1	121 (n= 1)	-	-	-	121	1	121 (n= 1)
A1 III6 o	3764	2	3764 (n= 4)	121 (n= 1)	-	-	121	1	121 (n= 5)
A1 III6 ν	3764	1	3764 (n= 1)	-	-	-	121	1	121 (n= 1)
A1 III6 α	121	1	121 (n= 1)	-	-	-	121	1	121 (n= 1)
A1 III6 δ	121	1	121 (n= 5)	-	-	-	121	1	121 (n= 5)
A1 III6 ε	121	1	121 (n= 1)	-	-	-	121	1	121 (n= 1)
A1 III6 η	3764	1	3764 (n= 3)	-	-	-	121	1	121 (n= 3)
A1 III6 κ	3764	1	3764 (n= 1)	-	-	-	121	1	121 (n= 1)
A1 III6 λ	121	1	121 (n= 4)	-	-	-	121	1	121 (n= 4)
A1 VII1 δ	121	1	121 (n= 1)	-	-	-	121	1	121 (n= 1)
A3 II1 κ	7763	1	7763 (n= 1)	-	-	-	121	1	121 (n= 1)

GENOTYPE	EXPECTED ST	n Observed STs	1st Observed ST	2nd Observed ST	3rd Observed ST	4th Observed ST	EXPECTED CC	n Observed CCs	Observed CC
A4 II1 δ	121	1	121 (n= 1)	-	-	-	121	1	121 (n= 1)
B1 I1 α	96	1	96 (n= 1)	-	-	-	96	1	96 (n= 1)
B1 I1 β	2855	1	2855 (n= 2)	-	-	-	96	1	96 (n= 2)
B1 I11 β	8008	1	8008 (n= 2)	-	-	-	96	1	96 (n= 2)
B1 I7 α	96	2	96 (n= 2)	8008 (n= 1)	-	-	96	1	96 (n= 3)
B1 I7 β	2855	1	2855 (n= 1)	-	-	-	96	1	96 (n= 1)
B1 II1 o	7763	1	7763 (n= 1)	-	-	-	121	1	121 (n= 1)
B1 II6 β	2855	1	2855 (n= 1)	-	-	-	96	1	96 (n= 1)
B1 IV1 α	2855	1	2855 (n= 6)	-	-	-	96	1	96 (n= 6)
B1 IV1 β	2855	1	2855 (n= 7)	-	-	-	96	1	96 (n= 7)
B1 IV1 γ	2855	1	2855 (n= 1)	-	-	-	96	1	96 (n= 1)
B1 IV1 δ	2855	1	2855 (n= 1)	-	-	-	96	1	96 (n= 1)
B1 IV10 β	96	1	96 (n= 1)	-	-	-	96	1	96 (n= 1)
B1 IV11 α	2855	2	2855 (n= 1)	7855 (n= 1)	-	-	96	1	96 (n= 2)
B1 IV11 γ	96	1	96 (n= 1)	-	-	-	96	1	96 (n= 1)
B1 IV11 η	96	1	96 (n= 1)	-	-	-	96	1	96 (n= 1)
B1 IV11 κ	96	1	96 (n= 1)	-	-	-	96	1	96 (n= 1)
B1 IV2 i	96	1	96 (n= 1)	-	-	-	96	1	96 (n= 1)
B1 IV2 α	2855	3	2855 (n= 7)	8008 (n= 1)	96 (n= 1)	-	96	1	96 (n= 9)
B1 IV2 β	2855	4	2855 (n= 12)	5001 (n= 1)	8008 (n= 1)	96 (n= 1)	96	1	96 (n= 15)
B1 IV2 γ	2855	2	2855 (n= 1)	96 (n= 1)	-	-	96	1	96 (n= 2)
B1 IV2 δ	2855	1	2855 (n= 1)	-	-	-	96	1	96 (n= 1)
B1 IV2 θ	8010	1	8010 (n= 1)	-	-	-	96	1	96 (n= 1)
B1 IV2 κ	96	1	96 (n= 1)	-	-	-	96	1	96 (n= 1)
B1 IV2 λ	96	1	96 (n= 1)	-	-	-	96	1	96 (n= 1)
B1 IV3 α	96	1	96 (n= 1)	-	-	-	96	1	96 (n= 1)
B1 IV3 β	2855	1	2855 (n= 1)	-	-	-	96	1	96 (n= 1)
B2 I1 ζ	45	1	45 (n= 1)	-	-	-	45	1	45 (n= 1)

GENOTYPE	EXPECTED ST	n Observed STs	1st Observed ST	2nd Observed ST	3rd Observed ST	4th Observed ST	EXPECTED CC	n Observed CCs	Observed CC
B2 IV2 γ	146	1	146 (n= 1)	-	-	-	5	1	5 (n= 1)
B3 II6 γ	146	1	146 (n= 1)	-	-	-	5	1	5 (n= 1)
B3 IV2 β	146	1	146 (n= 1)	-	-	-	5	1	5 (n= 1)
B3 IV2 ε	146	1	146 (n= 2)	-	-	-	5	1	5 (n= 2)
B3 IV2 θ	146	1	146 (n= 3)	-	-	-	5	1	5 (n= 3)
B4 I11 γ	1945	1	1945 (n= 1)	-	-	-	130	1	130 (n= 1)
B4 II1 θ	4774	1	4774 (n= 1)	-	-	-	130	1	130 (n= 1)
B4 III6 κ	4774	1	4774 (n= 1)	-	-	-	130	1	130 (n= 1)
B4 IV1 η	4774	1	4774 (n= 1)	-	-	-	130	1	130 (n= 1)
B4 IV6 κ	4774	2	4774 (n= 3)	7853 (n= 1)	-	-	130	1	130 (n= 4)
C4 IV1 β	7875	1	7875 (n= 1)	-	-	-	8	1	8 (n= 1)
C4 IV2 γ	2951	1	2951 (n= 1)	-	-	-	8	1	8 (n= 1)
D1 IV1 α	5993	1	5993 (n= 1)	-	-	-	ST5993	1	ST5993 (n= 1)
D1 IV1 β	407	1	407 (n= 1)	-	-	-	8	1	8 (n= 1)
D1 IV2 i	407	1	407 (n= 1)	-	-	-	8	1	8 (n= 1)
D1 IV2 β	407	2	407 (n= 1)	7878 (n= 1)	-	-	8	1	8 (n= 2)
D2 II1 δ	121	1	121 (n= 2)	-	-	-	121	1	121 (n= 2)
D2 IV2 α	2855	1	2855 (n= 2)	-	-	-	96	1	96 (n= 2)
D4 IV2 γ	1	1	1 (n= 1)	-	-	-	1	1	1 (n= 1)
D4 V1 β	1	1	1 (n= 1)	-	-	-	1	1	1 (n= 1)
D4 V1 γ	1	1	1 (n= 2)	-	-	-	1	1	1 (n= 2)
D4 V1 δ	1	1	1 (n= 3)	-	-	-	1	1	1 (n= 3)
D4 V1 θ	1	1	1 (n= 1)	-	-	-	1	1	1 (n= 1)
D5 IV1 β	15	1	15 (n= 1)	-	-	-	15	1	15 (n= 1)
D5 IV2 γ	15	1	15 (n= 1)	-	-	-	15	1	15 (n= 1)
D8 IV11 α	2855	1	2855 (n= 1)	-	-	-	96	1	96 (n= 1)
F1 II1 δ	121	1	121 (n= 1)	-	-	-	121	1	121 (n= 1)
F2 I12 α	425	1	425 (n= 1)	-	-	-	ST425	1	ST425 (n= 1)

GENOTYPE	EXPECTED ST	n Observed STs	1st Observed ST	2nd Observed ST	3rd Observed ST	4th Observed ST	EXPECTED CC	n Observed CCs	Observed CC
F2 IV9 α	8144	1	8144 (n= 2)	-	-	-	ST425	1	ST425 (n= 2)
F3 II1 δ	121	1	121 (n= 1)	-	-	-	121	1	121 (n= 1)
F7 I6 o	398	1	398 (n= 1)	-	-	-	398	1	398 (n= 1)
F7 I6 α	398	1	398 (n= 2)	-	-	-	398	1	398 (n= 2)
F7 I6 β	398	1	398 (n= 1)	-	-	-	398	1	398 (n= 1)
F7 I6 ζ	398	1	398 (n= 2)	-	-	-	398	1	398 (n= 2)
F7 I6 η	398	1	398 (n= 1)	-	-	-	398	1	398 (n= 1)
F7 I6 κ	398	1	398 (n= 2)	-	-	-	398	1	398 (n= 2)
F7 IV4 δ	7854	1	7854 (n= 1)	-	-	-	398	1	398 (n= 1)
F8 I6 δ	7877	1	7877 (n= 1)	-	-	-	398	1	398 (n= 1)
