# Supplementary material for: Staphylococcus aureus characterization in commercial rabbit farms reveals high genetic diversity and widespread antimicrobial resistance
Source: Front Vet Sci. 2025 Oct 30;12:1673809. doi: 10.3389/fvets.2025.1673809 (PMC12613231; doi:10.3389/fvets.2025.1673809)
Supplement: Supplementary file 4 [file Data_Sheet_4.pdf]

Additional File 4. Sequence types (ST) and clonal complexes (CC) identified per sampled farm.

FARMS	nSAMPLES	nST	ST1	ST2	ST3	ST4	nCC	CC1	CC2	CC3	CC4
FARM 1	12	3	96	121	3764	-	2	96	121	-	-
FARM 2	31	1	3764	-	-	-	1	121	-	-	-
FARM 3	2	1	4774	-	-	-	1	130	-	-	-
FARM 4	2	1	Unknown	-	-	-	1	Unknown	-	-	-
FARM 5	3	1	3764	-	-	-	1	121	-	-	-
FARM 6	3	1	4774	-	-	-	1	130	-	-	-
FARM 7	3	1	7853	-	-	-	1	130	-	-	-
FARM 8	4	1	121	-	-	-	1	121	-	-	-
FARM 9	2	1	3764	-	-	-	1	121	-	-	-
FARM 10	1	1	3764	-	-	-	1	121	-	-	-
FARM 11	2	1	3764	-	-	-	1	121	-	-	-
FARM 12	2	1	2855	-	-	-	1	96	-	-	-
FARM 13	1	1	2855	-	-	-	1	96	-	-	-
FARM 14	2	1	3764	-	-	-	1	121	-	-	-
FARM 15	3	2	121	2855	-	-	2	121	96	-	-
FARM 16	5	1	121	-	-	-	1	121	-	-	-
FARM 17	7	3	3764	8003	121	-	1	121	-	-	-
FARM 18	1	1	3764	-	-	-	1	121	-	-	-
FARM 19	3	2	121	96	-	-	2	121	96	-	-
FARM 20	2	2	3764	2855	-	-	2	96	121	-	-
FARM 21	2	2	3764	121	-	-	1	121	-	-	-
FARM 22	1	1	2855	-	-	-	1	96	-	-	-
FARM 23	2	2	121	2855	-	-	2	121	96	-	-
FARM 24	5	1	3764	-	-	-	1	121	-	-	-
FARM 25	2	1	121	-	-	-	1	121	-	-	-
FARM 26	4	2	8010	2855	-	-	1	96	-	-	-
FARM 27	6	2	3764	2855	-	-	2	121	96	-	-
FARM 28	1	1	121	-	-	-	1	121	-	-	-
FARM 29	3	1	121	-	-	-	1	121	-	-	-

FARMS	nSAMPLES	nST	ST1	ST2	ST3	ST4	nCC	CC1	CC2	CC3	CC4
FARM 30	1	1	2855	-	-	-	1	96	-	-	-
FARM 31	2	1	1	-	-	-	1	1	-	-	-
FARM 32	1	1	121	-	-	-	1	121	-	-	-
FARM 33	5	2	3764	2855	-	-	2	121	96	-	-
FARM 34	3	2	7876	121	-	-	1	121	-	-	-
FARM 35	8	3	121	96	8008	-	2	121	96	-	-
FARM 36	2	1	3764	-	-	-	1	121	-	-	-
FARM 37	1	1	2855	-	-	-	1	96	-	-	-
FARM 38	2	1	2855	-	-	-	1	96	-	-	-
FARM 39	1	1	2855	-	-	-	1	96	-	-	-
FARM 40	4	1	121	-	-	-	1	121	-	-	-
FARM 41	1	1	8009	-	-	-	1	121	-	-	-
FARM 42	2	2	2855	121	-	-	2	96	121	-	-
FARM 43	1	1	2855	-	-	-	1	96	-	-	-
FARM 44	3	1	3764	-	-	-	1	121	-	-	-
FARM 45	1	1	3764	-	-	-	1	121	-	-	-
FARM 46	1	1	398	-	-	-	1	398	-	-	-
FARM 47	1	1	3764	-	-	-	1	121	-	-	-
FARM 48	3	1	121	-	-	-	1	121	-	-	-
FARM 49	12	2	3764	8008	-	-	2	121	96	-	-
FARM 50	7	1	8757	-	-	-	1	121	-	-	-
FARM 51	2	2	3764	121	-	-	1	121	-	-	-
FARM 52	2	1	2855	-	-	-	1	96	-	-	-
FARM 53	1	1	2855	-	-	-	1	96	-	-	-
FARM 54	1	1	3764	-	-	-	1	121	-	-	-
FARM 55	1	1	7763	-	-	-	1	121	-	-	-
FARM 56	1	1	3764	-	-	-	1	121	-	-	-
FARM 57	2	1	121	-	-	-	1	121	-	-	-
FARM 58	2	1	121	-	-	-	1	121	-	-	-
FARM 59	1	1	2855	-	-	-	1	96	-	-	-

FARMS	nSAMPLES	nST	ST1	ST2	ST3	ST4	nCC	CC1	CC2	CC3	CC4
FARM 60	2	1	121	-	-	-	1	121	-	-	-
FARM 61	2	1	121	-	-	-	1	121	-	-	-
FARM 62	2	1	3764	-	-	-	1	121	-	-	-
FARM 63	1	1	4774	-	-	-	1	130	-	-	-
FARM 64	2	1	3764	-	-	-	1	121	-	-	-
FARM 65	1	1	121	-	-	-	1	121	-	-	-
FARM 66	1	1	3764	-	-	-	1	121	-	-	-
FARM 67	1	1	1	-	-	-	1	1	-	-	-
FARM 68	1	1	1	-	-	-	1	1	-	-	-
FARM 69	1	1	2855	-	-	-	1	96	-	-	-
FARM 70	3	1	2855	-	-	-	1	96	-	-	-
FARM 71	3	2	47	7878	-	-	1	8	-	-	-
FARM 72	2	2	2855	3764	-	-	2	96	121	-	-
FARM 73	3	1	3764	-	-	-	1	121	-	-	-
FARM 74	2	2	121	7854	-	-	2	121	398	-	-
FARM 75	2	1	3764	-	-	-	1	121	-	-	-
FARM 76	1	1	121	-	-	-	1	121	-	-	-
FARM 77	3	1	3764	-	-	-	1	121	-	-	-
FARM 78	2	1	3764	-	-	-	1	121	-	-	-
FARM 79	1	1	3764	-	-	-	1	121	-	-	-
FARM 80	2	1	3764	-	-	-	1	121	-	-	-
FARM 81	1	1	121	-	-	-	1	121	-	-	-
FARM 82	1	1	8727	-	-	-	1	121	-	-	-
FARM 83	2	1	121	-	-	-	1	121	-	-	-
FARM 84	1	1	8727	-	-	-	1	121	-	-	-
FARM 85	1	1	121	-	-	-	1	121	-	-	-
FARM 86	1	1	3764	-	-	-	1	121	-	-	-
FARM 87	1	1	3764	-	-	-	1	121	-	-	-
FARM 88	2	1	3764	-	-	-	1	121	-	-	-
FARM 89	3	1	2855	-	-	-	1	96	-	-	-

FARMS	nSAMPLES	nST	ST1	ST2	ST3	ST4	nCC	CC1	CC2	CC3	CC4
FARM 90	1	1	2855	-	-	-	1	96	-	-	-
FARM 91	2	1	3764	-	-	-	1	121	-	-	-
FARM 92	2	1	3764	-	-	-	1	121	-	-	-
FARM 93	1	1	3764	-	-	-	1	121	-	-	-
FARM 94	1	1	3764	-	-	-	1	121	-	-	-
FARM 95	2	2	3764	398	-	-	2	121	398	-	-
FARM 96	1	1	2855	-	-	-	1	96	-	-	-
FARM 97	2	1	121	-	-	-	1	121	-	-	-
FARM 98	1	1	3764	-	-	-	1	121	-	-	-
FARM 99	1	1	3764	-	-	-	1	121	-	-	-
FARM 100	1	1	3764	-	-	-	1	121	-	-	-
FARM 101	1	1	3764	-	-	-	1	121	-	-	-
FARM 102	1	1	3764	-	-	-	1	121	-	-	-
FARM 103	1	1	3764	-	-	-	1	121	-	-	-
FARM 104	1	1	3764	-	-	-	1	121	-	-	-
FARM 105	1	1	3764	-	-	-	1	121	-	-	-
FARM 106	1	1	2855	-	-	-	1	96	-	-	-
FARM 107	1	1	121	-	-	-	1	121	-	-	-
FARM 108	1	1	121	-	-	-	1	121	-	-	-
FARM 109	2	2	2855	121	-	-	2	96	121	-	-
FARM 110	1	1	146	-	-	-	1	5	-	-	-
FARM 111	1	1	121	-	-	-	1	121	-	-	-
FARM 112	2	1	121	-	-	-	1	121	-	-	-
FARM 113	2	2	45	96	-	-	2	45	96	-	-
FARM 114	1	1	121	-	-	-	1	121	-	-	-
FARM 115	1	1	121	-	-	-	1	121	-	-	-
FARM 116	1	1	3764	-	-	-	1	121	-	-	-
FARM 117	1	1	3764	-	-	-	1	121	-	-	-
FARM 118	2	1	3764	-	-	-	1	121	-	-	-
FARM 119	1	1	121	-	-	-	1	121	-	-	-

FARMS	nSAMPLES	nST	ST1	ST2	ST3	ST4	nCC	CC1	CC2	CC3	CC4
FARM 120	2	1	2855	-	-	-	1	96	-	-	-
FARM 121	1	1	3764	-	-	-	1	121	-	-	-
FARM 122	2	2	15	2855	-	-	2	15	96	-	-
FARM 123	1	1	121	-	-	-	1	121	-	-	-
FARM 124	2	1	2855	-	-	-	1	96	-	-	-
FARM 125	2	1	121	-	-	-	1	121	-	-	-
FARM 126	2	2	3764	398	-	-	2	121	398	-	-
FARM 127	2	1	3764	-	-	-	1	121	-	-	-
FARM 128	1	1	121	-	-	-	1	121	-	-	-
FARM 129	2	2	7855	3764	-	-	2	96	121	-	-
FARM 130	5	3	2855	8144	Unknown	-	3	425	96	Unknown	-
FARM 131	2	2	3764	398	-	-	2	121	398	-	-
FARM 132	4	2	7877	121	-	-	2	398	121	-	-
FARM 133	3	2	3764	121	-	-	1	121	-	-	-
FARM 134	1	1	121	-	-	-	1	121	-	-	-
FARM 135	1	1	3764	-	-	-	1	121	-	-	-
FARM 136	1	1	121	-	-	-	1	121	-	-	-
FARM 137	1	1	3764	-	-	-	1	121	-	-	-
FARM 138	2	2	15	2855	-	-	2	15	96	-	-
FARM 139	1	1	146	-	-	-	1	5	-	-	-
FARM 140	2	1	2855	-	-	-	1	96	-	-	-
FARM 141	2	1	4774	-	-	-	1	130	-	-	-
FARM 142	1	1	398	-	-	-	1	398	-	-	-
FARM 143	1	1	121	-	-	-	1	121	-	-	-
FARM 144	2	2	3764	121	-	-	1	121	-	-	-
FARM 145	1	1	96	-	-	-	1	96	-	-	-
FARM 146	1	1	121	-	-	-	1	121	-	-	-
FARM 147	2	1	3764	-	-	-	1	121	-	-	-
FARM 148	1	1	3764	-	-	-	1	121	-	-	-
FARM 149	1	1	121	-	-	-	1	121	-	-	-

FARMS	nSAMPLES	nST	ST1	ST2	ST3	ST4	nCC	CC1	CC2	CC3	CC4
FARM 150	1	1	121	-	-	-	1	121	-	-	-
FARM 151	1	1	121	-	-	-	1	121	-	-	-
FARM 152	1	1	121	-	-	-	1	121	-	-	-
FARM 153	1	1	121	-	-	-	1	121	-	-	-
FARM 154	1	1	3764	-	-	-	1	121	-	-	-
FARM 155	3	1	3764	-	-	-	1	121	-	-	-
FARM 156	1	1	3764	-	-	-	1	121	-	-	-
FARM 157	1	1	2855	-	-	-	1	96	-	-	-
FARM 158	1	1	3764	-	-	-	1	121	-	-	-
FARM 159	3	2	121	398	-	-	2	121	398	-	-
FARM 160	1	1	398	-	-	-	1	398	-	-	-
FARM 161	1	1	3764	-	-	-	1	121	-	-	-
FARM 162	1	1	121	-	-	-	1	121	-	-	-
FARM 163	1	1	2855	-	-	-	1	96	-	-	-
FARM 164	1	1	3764	-	-	-	1	121	-	-	-
FARM 165	1	1	2951	-	-	-	1	8	-	-	-
FARM 166	2	1	121	-	-	-	1	121	-	-	-
FARM 167	2	1	121	-	-	-	1	121	-	-	-
FARM 168	1	1	121	-	-	-	1	121	-	-	-
FARM 169	1	1	3764	-	-	-	1	121	-	-	-
FARM 170	1	1	121	-	-	-	1	121	-	-	-
FARM 171	1	1	121	-	-	-	1	121	-	-	-
FARM 172	1	1	121	-	-	-	1	121	-	-	-
FARM 173	1	1	146	-	-	-	1	5	-	-	-
FARM 174	1	1	3764	-	-	-	1	121	-	-	-
FARM 175	1	1	398	-	-	-	1	398	-	-	-
FARM 176	1	1	121	-	-	-	1	121	-	-	-
FARM 177	1	1	2855	-	-	-	1	96	-	-	-
FARM 178	1	1	121	-	-	-	1	121	-	-	-
FARM 179	2	1	121	-	-	-	1	121	-	-	-

FARMS	nSAMPLES	nST	ST1	ST2	ST3	ST4	nCC	CC1	CC2	CC3	CC4
FARM 180	1	1	121	-	-	-	1	121	-	-	-
FARM 181	1	1	121	-	-	-	1	121	-	-	-
FARM 182	1	1	121	-	-	-	1	121	-	-	-
FARM 183	1	1	3764	-	-	-	1	121	-	-	-
FARM 184	1	1	2855	-	-	-	1	96	-	-	-
FARM 185	1	1	3764	-	-	-	1	121	-	-	-
FARM 186	1	1	121	-	-	-	1	121	-	-	-
FARM 187	1	1	121	-	-	-	1	121	-	-	-
FARM 188	1	1	3764	-	-	-	1	121	-	-	-
FARM 189	1	1	3764	-	-	-	1	121	-	-	-
FARM 190	1	1	3764	-	-	-	1	121	-	-	-
FARM 191	2	2	3764	121	-	-	1	121	-	-	-
FARM 192	1	1	121	-	-	-	1	121	-	-	-
FARM 193	1	1	3764	-	-	-	1	121	-	-	-
FARM 194	3	1	121	-	-	-	1	121	-	-	-
FARM 195	2	2	121	146	-	-	2	121	5	-	-
FARM 196	5	2	96	2855	-	-	1	96	-	-	-
FARM 197	2	1	2855	-	-	-	1	96	-	-	-
FARM 198	3	1	3764	-	-	-	1	121	-	-	-
FARM 199	1	1	3764	-	-	-	1	121	-	-	-
FARM 200	2	1	3764	-	-	-	1	121	-	-	-
FARM 201	1	1	398	-	-	-	1	398	-	-	-
FARM 202	1	1	121	-	-	-	1	121	-	-	-
FARM 203	1	1	3764	-	-	-	1	121	-	-	-
FARM 204	1	1	8727	-	-	-	1	121	-	-	-
FARM 205	1	1	3764	-	-	-	1	121	-	-	-
FARM 206	1	1	1	-	-	-	1	1	-	-	-
FARM 207	1	1	121	-	-	-	1	121	-	-	-
FARM 208	1	1	121	-	-	-	1	121	-	-	-
FARM 209	1	1	121	-	-	-	1	121	-	-	-

FARMS	nSAMPLES	nST	ST1	ST2	ST3	ST4	nCC	CC1	CC2	CC3	CC4
FARM 210	1	1	3764	-	-	-	1	121	-	-	-
FARM 211	1	1	121	-	-	-	1	121	-	-	-
FARM 212	1	1	3764	-	-	-	1	121	-	-	-
FARM 213	1	1	3764	-	-	-	1	121	-	-	-
FARM 214	2	1	3764	-	-	-	1	121	-	-	-
FARM 215	2	1	121	-	-	-	1	121	-	-	-
FARM 216	1	1	3764	-	-	-	1	121	-	-	-
FARM 217	1	1	3764	-	-	-	1	121	-	-	-
FARM 218	1	1	8760	-	-	-	1	96	-	-	-
FARM 219	1	1	3764	-	-	-	1	121	-	-	-
FARM 220	1	1	121	-	-	-	1	121	-	-	-
FARM 221	2	1	121	-	-	-	1	121	-	-	-
FARM 222	1	1	3764	-	-	-	1	121	-	-	-
FARM 223	1	1	2855	-	-	-	1	96	-	-	-
FARM 224	1	1	121	-	-	-	1	121	-	-	-
FARM 225	2	1	3764	-	-	-	1	121	-	-	-
FARM 226	1	1	3764	-	-	-	1	121	-	-	-
FARM 227	4	3	121	146	5001	-	3	121	5	96	-
FARM 228	2	1	3764	-	-	-	1	121	-	-	-
FARM 229	2	1	1	-	-	-	1	1	-	-	-
FARM 230	4	1	146	-	-	-	1	5	-	-	-
FARM 231	5	1	2855	-	-	-	1	96	-	-	-
FARM 232	4	2	3764	146	-	-	2	121	5	-	-
FARM 233	3	1	121	-	-	-	1	121	-	-	-
FARM 234	4	2	121	2855	-	-	2	121	96	-	-
FARM 235	2	2	3764	Unknown	-	-	2	121	Unknown	-	-
FARM 236	2	1	3764	-	-	-	1	121	-	-	-
FARM 237	4	1	2855	-	-	-	1	96	-	-	-
FARM 238	1	1	121	-	-	-	1	121	-	-	-
FARM 239	2	1	3764	-	-	-	1	121	-	-	-

FARMS	nSAMPLES	nST	ST1	ST2	ST3	ST4	nCC	CC1	CC2	CC3	CC4
FARM 240	3	1	3764	-	-	-	1	121	-	-	-
FARM 241	4	1	3764	-	-	-	1	121	-	-	-
FARM 242	4	1	3764	-	-	-	1	121	-	-	-
FARM 243	4	1	3764	-	-	-	1	121	-	-	-
FARM 244	5	1	8759	-	-	-	1	96	-	-	-
FARM 245	5	1	2855	-	-	-	1	96	-	-	-
FARM 246	1	1	3764	-	-	-	1	121	-	-	-
FARM 247	6	1	3764	-	-	-	1	121	-	-	-
FARM 248	3	1	2855	-	-	-	1	96	-	-	-
FARM 249	4	1	3764	-	-	-	1	121	-	-	-
FARM 250	3	1	121	-	-	-	1	121	-	-	-
FARM 251	3	1	3764	-	-	-	1	121	-	-	-
FARM 252	5	1	121	-	-	-	1	121	-	-	-
FARM 253	4	1	8758	-	-	-	1	96	-	-	-
FARM 254	1	1	15	-	-	-	1	15	-	-	-
FARM 255	5	2	2855	121	-	-	2	96	121	-	-
FARM 256	2	1	121	-	-	-	1	121	-	-	-
FARM 257	1	1	121	-	-	-	1	121	-	-	-
FARM 258	2	1	1	-	-	-	1	1	-	-	-
FARM 259	1	1	121	-	-	-	1	121	-	-	-
FARM 260	4	1	121	-	-	-	1	121	-	-	-
FARM 261	7	2	3764	121	-	-	1	121	-	-	-
FARM 262	4	1	2855	-	-	-	1	96	-	-	-
FARM 263	5	4	425	121	2855	Unknown	4	425	121	96	Unknown
FARM 264	1	1	121	-	-	-	1	121	-	-	-
FARM 265	8	2	3764	121	-	-	1	121	-	-	-
FARM 266	6	2	96	3764	-	-	2	96	121	-	-
FARM 267	2	2	121	2855	-	-	2	121	96	-	-
FARM 268	4	2	121	3764	-	-	1	121	-	-	-
FARM 269	3	1	121	-	-	-	1	121	-	-	-

FARMS	nSAMPLES	nST	ST1	ST2	ST3	ST4	nCC	CC1	CC2	CC3	CC4
FARM 270	2	1	3764	-	-	-	1	121	-	-	-
FARM 271	1	1	47	-	-	-	1	8	-	-	-
FARM 272	2	1	2855	-	-	-	1	96	-	-	-
FARM 273	4	2	2855	1945	-	-	2	130	96	-	-
FARM 274	12	3	3764	2855	96	-	2	121	96	-	-
FARM 275	4	2	146	121	-	-	2	5	121	-	-
FARM 276	2	1	96	-	-	-	1	96	-	-	-
FARM 277	5	1	3764	-	-	-	1	121	-	-	-
FARM 278	3	2	1	Unknown	-	-	2	96	1	-	-
FARM 279	3	1	96	-	-	-	1	96	-	-	-
FARM 280	4	2	7763	Unknown	-	-	2	121	Unknown	-	-
FARM 281	5	1	7763	-	-	-	1	121	-	-	-
FARM 282	3	1	8763	-	-	-	1	121	-	-	-
FARM 283	3	2	121	146	-	-	2	121	5	-	-
FARM 284	1	1	3764	-	-	-	1	121	-	-	-
FARM 285	1	1	121	-	-	-	1	121	-	-	-
