# Supplementary material for: Staphylococcus aureus characterization in commercial rabbit farms reveals high genetic diversity and widespread antimicrobial resistance
Source: Front Vet Sci. 2025 Oct 30;12:1673809. doi: 10.3389/fvets.2025.1673809 (PMC12613231; doi:10.3389/fvets.2025.1673809)
Supplement: Supplementary file 5 [file Data_Sheet_5.pdf]

Additional File 5. Number and frequency (%) of clonal complexes classified by the province of origin.

PROVINCES	n SAMPLES	n FARMS	CC121	CC96	CC398	CC5	CC1	CC8	CC15	CC130	CC425	CC45
León	78	21	70 (89.74)	5 (6.41)	1 (1.28)	2 (2.56)						
València/Valencia	38	10	29 (76.32)	9 (23.68)								
Gipuzkoa/Guipúzcoa	37	11	9 (24.32)	26 (70.27)							2 (5.41)	
Ourense	33	18	16 (48.48)	7 (21.21)				1 (3.03)		9 (27.27)		
Barcelona	32	19	22 (68.75)	2 (6.25)	3 (9.38)		4 (12.5)	1 (3.12)				
Castelló/Castellón	32	16	21 (65.62)	8 (25)			3 (9.38)					
Albacete	29	12	27 (93.1)	2 (6.9)								
Tarragona	28	15	15 (53.57)	1 (3.57)		9 (32.14)	3 (10.71)					
Pontevedra	28	12	16 (57.14)	11 (39.29)	1 (3.57)							
A Coruña	27	16	26 (96.3)	1 (3.7)								
Teruel	20	13	19 (95)	1 (5)								
Valladolid	19	10	13 (68.42)	3 (15.79)	2 (10.53)				1 (5.26)			
Bizkaia/Vizcaya	19	7	10 (52.63)	9 (47.37)								
Segovia	17	11	16 (94.12)		1 (5.88)							
Lugo	17	11	14 (82.35)	3 (17.65)								
Girona	14	5	5 (35.71)	5 (35.71)		1 (7.14)		3 (21.43)				
Zamora	14	9	13 (92.86)	1 (7.14)								
Palencia	14	4	14 (100)									
Lleida	13	6	12 (92.31)		1 (7.69)							
Huesca	11	6	8 (72.73)	3 (27.27)								
Cantabria	11	7	10 (90.91)	1 (9.09)								
Cuenca	10	5	6 (60)	1 (10)		1 (10)			1 (10)		1 (10)	
Zaragoza	10	3	9 (90)	1 (10)								
La Rioja	8	3	2 (25)	6 (75)								
Murcia	8	3	8 (100)									
Burgos	6	2	1 (16.67)	5 (83.33)								
Salamanca	6	3	4 (66.67)	2 (33.33)								

Badajoz	4	1		2 (50)			2 (50)	
Ávila	4	4	4 (100)					
Araba/Álava	4	1		4 (100)				
Soria	4	2		4 (100)				
Navarra	3	2	1 (33.33)	1 (33.33)				1 (33.33)
Santa Cruz De Tenerife	3	3	1 (33.33)			2 (66.67)		
Alacant/Alicante	3	1	3 (100)					
Cáceres	2	1	2 (100)					
Guadalajara	2	2	2 (100)					
Granada	1	1			1 (100)			
Jaén	1	1	1 (100)					
Málaga	1	1	1 (100)					
